# Embryonic Stem Cell-Derived Microvesicles Induce Gene Expression Changes in Müller Cells of the Retina

**DOI:** 10.1371/journal.pone.0050417

**Published:** 2012-11-30

**Authors:** Diana Katsman, Emma J. Stackpole, Daniel R. Domin, Debora B. Farber

**Affiliations:** 1 Jules Stein Eye Institute and Department of Ophthalmology, University of California Los Angeles, Los Angeles, California, United States of America; 2 Molecular Biology Institute, University of California Los Angeles, Los Angeles, California, United States of America; 3 Brain Research Institute, University of California Los Angeles, Los Angeles, California, United States of America; University of Florida, United States of America

## Abstract

Cell-derived microvesicles (MVs), recognized as important components of cell-cell communication, contain mRNAs, miRNAs, proteins and lipids and transfer their bioactive contents from parent cells to cells of other origins. We have studied the effect that MVs released from embryonic stem cells (ESMVs) have on retinal progenitor Müller cells. Cultured human Müller cells were exposed to mouse ESMVs every 48 hours for a total of 9 treatments. Morphological changes were observed by light microscopy in the treated cells, which grew as individual heterogeneous cells, compared to the uniform, spindle-like adherent cellular sheets of untreated cells. ESMVs transferred to Müller cells embryonic stem cell (ESC) mRNAs involved in the maintenance of pluripotency, including *Oct4* and *Sox2*, and the miRNAs of the 290 cluster, important regulators of the ESC-specific cell cycle. Moreover, ESMV exposure induced up-regulation of the basal levels of endogenous human *Oct4* mRNA in Müller cells. mRNA and miRNA microarrays of ESMV-treated vs. untreated Müller cells revealed the up-regulation of genes and miRNAs involved in the induction of pluripotency, cellular proliferation, early ocular genes and genes important for retinal protection and remodeling, as well as the down-regulation of inhibitory and scar-related genes and miRNAs involved in differentiation and cell cycle arrest. To further characterize the heterogeneous cell population of ESMV-treated Müller cells, their expression of retinal cell markers was compared to that in untreated control cells by immunocytochemistry. Markers for amacrine, ganglion and rod photoreceptors were present in treated but not in control Müller cells. Together, our findings indicate that ESMs induce de-differentiation and pluripotency in their target Müller cells, which may turn on an early retinogenic program of differentiation.

## Introduction

Microvesicles (MVs), a heterogeneous population of vesicles (30nm to 1μm in diameter) that are released into the intercellular environment by the majority of cells, are increasingly recognized as important components of cell-cell communication, and as being capable of exerting pleiotropic effects on the surrounding cells [1], [2]. MVs were first identified in human plasma [3], and until recently believed to be inert cellular debris. They are released either by direct budding from the cell plasma membrane as shedding vesicles or as exosomes that originate in the endosomal membrane compartment and are extruded from the cell surface of activated cells [1], [2], [4], [5]. Our group and others have demonstrated that MVs contain mRNA, miRNA, proteins and lipids and that they can transfer their contents to cells of other origins, acting as “physiologic liposomes” [6], [7]. MVs may affect their target cells by stimulating them directly via surface-expressed ligands, by transferring surface receptors between cells, or by transferring genetic information from the parent to the target cells [1], [7], [8]. MVs derived from adult progenitor cells of several tissues have been shown to induce not only stem cell differentiation along the lineages of the MV donor [8]–[10] but also the endogenous regenerative capacity of those tissues [11], helping to repopulate and repair, for example, injured liver [9], lung [10] and kidneys [1], [12].

Embryonic stem cell-derived MVs (ESMVs) are enriched in mRNAs for early transcription factors and miRNAs important for embryonic stem cell (ESC) pluripotency [6], [13]. ESMVs have been shown to promote the survival and improve the expansion of hematopoietic progenitor cells [7]. These findings suggest that by transfer of stem cell-specific molecules, ESMVs can induce the activation of endogenous, adult, quiescent progenitor cells increasing their pluripotency and possibly their ability to repair damaged tissues. While evidence of the protective and pro-regenerative effects of MVs on whole tissues is increasing, no data on the effects of ESMVs on individual progenitor cell populations has been obtained to date. In this study, we investigated the effects that ESMVs have on the morphology, mRNA and miRNA expression of cultured retinal progenitor Müller cells.

Müller cells, the analogues of radial glia in the retina [14], are emerging as likely candidates for retinal progenitor cells [15]–[18]. They meet several of the requirements to be considered progenitor cells, including the ability to differentiate along multiple retinal lineages such as photoreceptors and inner retina neurons [15]–[19]. Müller glia are the source of near complete retinal restoration in fish [20], and of limited retinal regeneration in chicken [15], [17], [21], [22]. Recently, there have been reports of Müller glial cell proliferation and differentiation into cells of retinal lineage in adult rodents after injury [16], [23]. While Müller cells are activated in the injured retina with some regenerative success, functional retinal recovery has not yet been achieved [24]. Identification of factors that induce Müller cells to de-differentiate, enter the cell cycle, and differentiate along retinal neural lineages may lead to novel therapy development for retinal degenerative diseases. Our study explored the tantalizing possibility of employing ESMVs as agents that activate the regeneration program in Müller cells. Here we demonstrate that ESMVs added to the cultures of retinal progenitor Müller cells induce in them morphological changes towards a more de-differentiated phenotype and cause selective transfer of ESC mRNA and miRNA, resulting in induction of embryonic and early retinal genes. We also show that ESMV treatment induced a transcriptome change in Müller cells, suggestive of de-differentiation and activation of a retinal regeneration program. We observed the up-regulation of pluripotency and early retinal genes, genes involved in retinal protection and inducers of retinal regeneration, as well as multiple extracellular matrix (ECM) modifying molecules that create a permissive environment for retinal regeneration, and down-regulation of genes promoting differentiation and inhibitory ECM and scar components. Moreover, we show that ESMVs induced a shift in the miRNA transcriptome of Müller cells towards a de-differentiated progenitor state. Together, these results suggest that ESMVs hold future promise as therapeutic agents to activate the retina's endogenous regenerative potential.

## Results

### ESMV treatment induces morphological changes in Müller cells in culture

**Figure 1:**
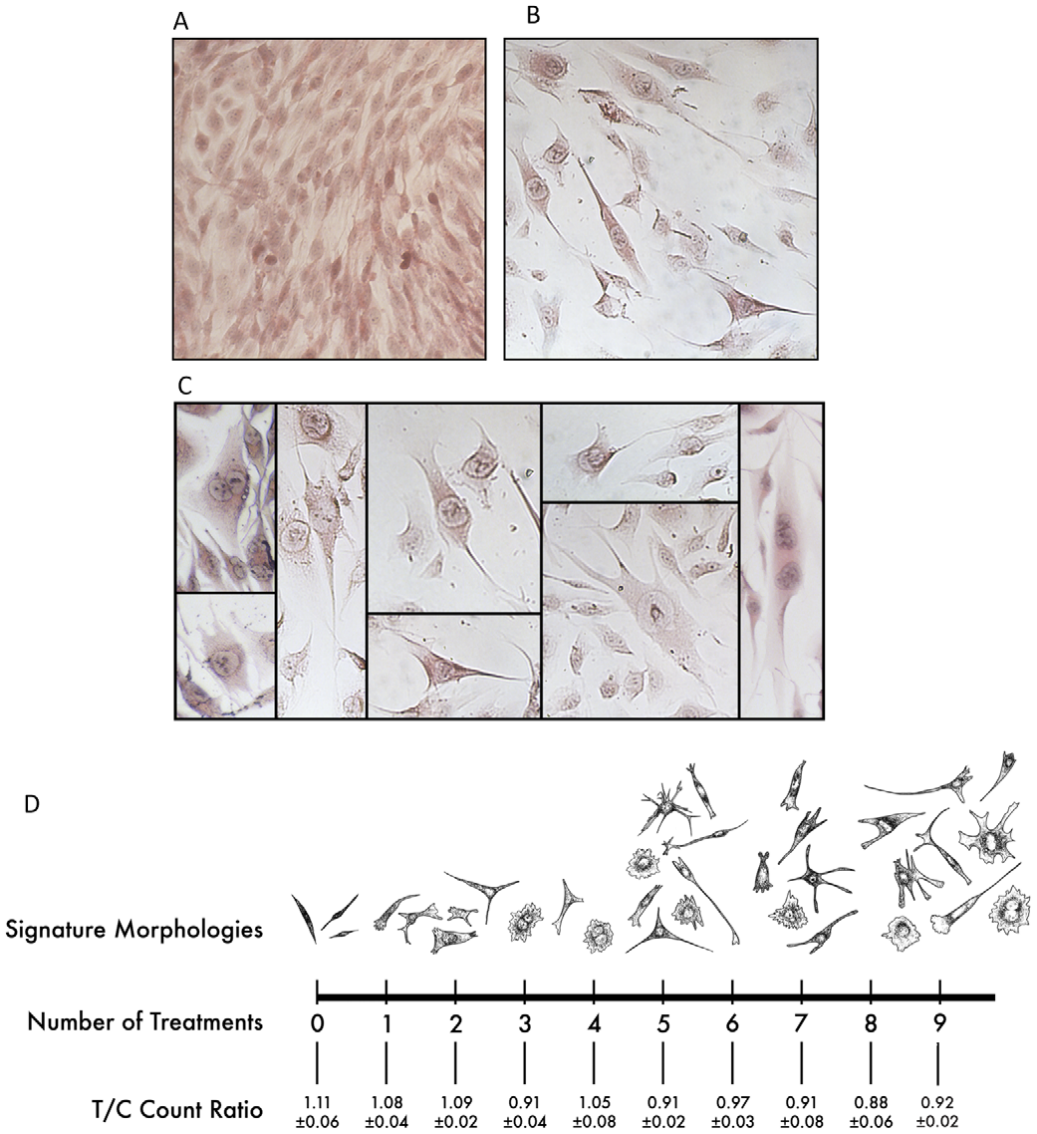
ESMVs induce morphological changes in Müller cells. (A) Untreated Müller cells growing as homogeneous, bipolar, spindle-like adherent cell “sheets” (B) Müller cells post 9 ESMV treatments, growing as morphologically heterogeneous individual cells, some with multiple cellular processes, others with enlarged nuclei or multinucleated, many having visible metaphase plates and numerous stellar-shaped. (C) Collage of individual cells morphologically unique to the ESMV treatment group. (D) Timeline of the morphological changes that take place in Müller cells after ESMV treatments. As Müller cells cultures progressively received more ESMV treatments, cells with apical boutons and varied morphologies appeared. The control group did not exhibit morphological changes, remaining a uniform culture of homogeneous bipolar cell sheets. ESMV-treated (T) and control (C) cells were counted after each treatment and the ratio of treated to control cells calculated (T/C ± S.E.M.).

ESMVs isolated from the supernatant of cultured mouse ESCs were added to human Müller cell cultures every 48 hours for a total of 9 treatments. Although the treated and non-treated (control) cultures were initiated from the same passage, number of cells, and confluence level of Müller cells, morphological differences became evident between control and ESMV-exposed cells as early as after the first treatment (Figure 1). In contrast to control cells that grew as uniform, spindle-like, adherent cellular sheets characteristic of typical Müller cells cultures (Figure 1A), as ESMV treatments progressed, the exposed Müller cells increasingly grew as individual heterogeneous cells, demonstrating decreased cell-cell adhesion, presence of cells with multiple processes, stellate cells, multinucleated cells, and cells with unilateral boutons and extensive processes (Figures 1 B, and C). Often, the nuclei of ESMV-treated cells were enlarged, many demonstrating visible metaphase plates. Cell count comparison between ESMV-treated and control cultures did not reveal significant decline in the overall number of cells in the treatment group (Figure 1D).

### ESMVs selectively transfer to Müller cells mRNA and miRNA transcripts known to induce pluripotency and the expression of embryonic and early retinal genes

**Figure 2:**
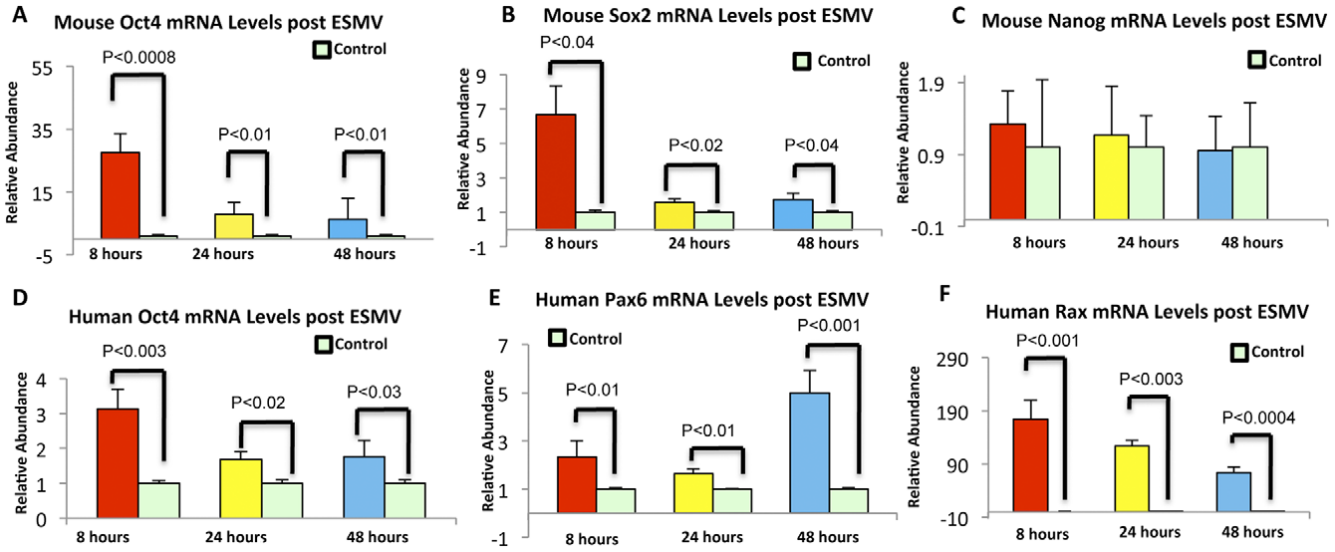
ESMVs transfer embryonic stem cell mRNA to Müller cells and induce the expression of endogenous embryonic transcripts in Müller cells. Using qRT-PCR, embryonic stem cell-specific mRNA transfer was assayed with mouse-specific *Oct4* and *Sox2* primers, while induction of endogenous Müller cell genes was detected using human *Oct4*, *Pax6*, and *Rax* primers. Bar plots show the fold change in expression for the embryonic stem cell-specific [*Oct4* (A), (D) and *Sox2* (B)] and early retinal [(*Pax6* (E) and *Rax* (F)] transcripts in Müller cells exposed for 8, 24, and 48 hours to ESMVs, compared to those in control Müller cells that received medium changes only (light green bars). No *Nanog* transfer was observed (C). *Gapdh* was used as a loading control for qRT-PCR. Error bars represent S.E.M. Student's t-test, performed to assay the difference between experimental and control Müller cells groups, showed that these differences were significant, all of them with p-values<0.05, except for *Nanog* mRNA (C).

We had previously demonstrated the presence of pluripotency factors such as the mRNAs encoding Oct4, Nanog and Gata-4 as well as miR-292, -294 and -295 in mouse ESMVs using qRT-PCR [6]. We then examined the level of these transcripts as well as of *Sox2, Klf4* and *Lin28* mRNAs in 4 independent ESMV populations and found that they are abundant and present in comparable amounts (unpublished results). We now investigated whether ESMVs transfer these pluripotency-inducing mRNAs and miRNAs to Müller cells and whether their expression as well as that of embryonic and early retinal genes is altered within Müller cells at 8, 24, and 48 hours post-ESMV exposure. With the use of qRT-PCR and species-specific primers we were able to distinguish between the transfer by ESMVs of mRNA transcripts from mouse ESCs and the induction by ESMVs of endogenous transcripts of human Müller cells. While mouse *Oct4* and *Sox2* mRNAs were transferred from ESMVs and remained elevated in Müller cells 48 hours after ESMV treatment (Figure 2, A and B), no Nanog transfer was observed at any time point post-treatment (Figure 2C), suggesting that ESMVs transfer genetic information by a selective mechanism.

Human *Oct4* mRNA in ESMV-treated Müller cells was increased 3-fold as early as 8 hours post-ESMV exposure and remained elevated for the next 40 hours (Figure 2D), indicating that the induction of the endogenous *Oct4* mRNA of Müller cells by ESMVs begins shortly after exposure and persists for days. The levels of *Pax6* and *Rax* mRNAs, which encode transcription factors known to be expressed throughout retinogenesis by multipotent retinal progenitor cells [25], [26], were found elevated 8 hours post-ESMV exposure and persisted at the 48 hour time point (Figure 2, E and F).

That the results above were specific to the ESMV treatment was corroborated by using two other controls in addition to the Müller cells that had not been exposed to ESMVs: a) the residue from equal volume of ESGRO Complete PLUS medium as that used in the isolation of ESMVs from ESCs, after ultracentrifugation at 200,000 g for 3.5 hours, was re-suspended in Müller cell culture medium and then incubated with Müller cell cultures for 8, 24 or 48 hours to determine whether it could modify the expression of the specific mRNAs; and b) The same volume of medium from a culture of mouse embryonic fibroblasts (MEFs) as that used in the isolation of ESMVs from ESCs was processed as in a) to see if microvesicles released by cells different from ESCs could also change the expression of the studied mRNAs. After qRT-PCR with the specific primers, the levels for mouse *Oct4* and *Sox2* as well as human *Oct4, Pax6* and *Rax* mRNAs were the same in Müller cells not exposed to ESMVs and in Müller cells treated with the ESGRO medium components or with the MEF conditioned medium. These results demonstrate that only incubation with ESMVs changes the specific mRNA levels in Müller cells.

**Figure 3:**
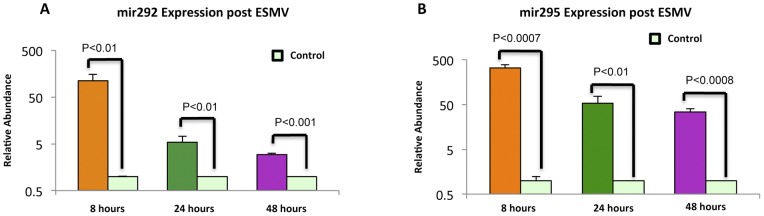
ESMVs transfer ESC miRNA to Müller cells. Bar plots showing the fold change in the levels of ESC-specific miRNA 292 (A) and miRNA 295 (B) in Müller cells at 8, 24, and 48 hours after ESMV treatment. The levels of both miRNAs tested were persistently elevated at 48 hours post transfer, indicating that these miRNAs are not degraded for days after transfer. The y-axis correspond to the fold changes between the treatment and control (light green) groups of Müller cells. Error bars represent S.E.M. Significant differences between experimental and control groups were determined by Student's t-test. All p-values were <0.01.

miRNAs, small noncoding RNAs, are important regulators of gene expression and maintenance of ESC pluripotency and cell fate determination [27]–[29]. It has been demonstrated that ESMVs are highly enriched in miRNAs [6], including ESC-specific miRNAs of the 290-cluster, known to be involved in maintenance of ESC pluripotency [28]. We hypothesized that the transfer of miRNAs is likely to be one of the mechanisms by which ESMVs influence gene expression in Müller cells. Using qRT-PCR, we tested for the presence of miRNA-292 and -295 in Müller cells at 8, 24, and 48 hours post-ESMV treatment, using U6 snRNA, a small nuclear RNA ubiquitous in mammalian cells, as normalizer. Since mature miRNA transcripts are very short, a strategy that uses a stem-loop RT primer was used [6]. To avoid confounding levels of such small transcripts by enrichment methods for ESMVs, we used the same total RNA samples used earlier for mRNA transfer studies to assay miRNA transfer. Both miRNA -292 (Figure 3A) and 295 (Figure 3B) were found to transfer efficiently to Müller cells and persist for 48 hours post-treatment, indicating that miRNAs are not degraded, possibly playing a role in gene expression alterations of Müller cells.

### Genome-wide analysis of gene expression changes induced in Müller cells by ESMV exposure

We compared the transcriptional response of Müller cells after 8, 24, and 48 hours of ESMV exposure with the transcriptome of control Müller cells cultured for the same number of hours by hybridization of cDNA from 3 (8 and 24 hours) and 2 (48 hours) independent biological samples of control and ESMV-treated Müller cells, each in triplicate, to Agilent human 8X60K cDNA arrays (Agilent, Santa Clara, CA, USA). The RMA algorithm was used for data normalization [30]. The minimum thresholds for selecting significant genes were set at ≥3 log_2_-transformed fold-change and FDR-corrected p<0.001. Genes that met both criteria simultaneously were considered significantly changed.

Known marker genes of Müller glia were detected in the microarrays [20], [31], [32]: glutamine synthetase (Glu1), clusterin (Clu), dickkopf homolog 3 (Dkk3), aquaporin 4 (Aqp4), S100 calcium binding protein A16, Apolipoprotein E (ApoE), Vimentin (VIM), and glial fibrillary acidic protein (GFAP).

**Figure 4:**
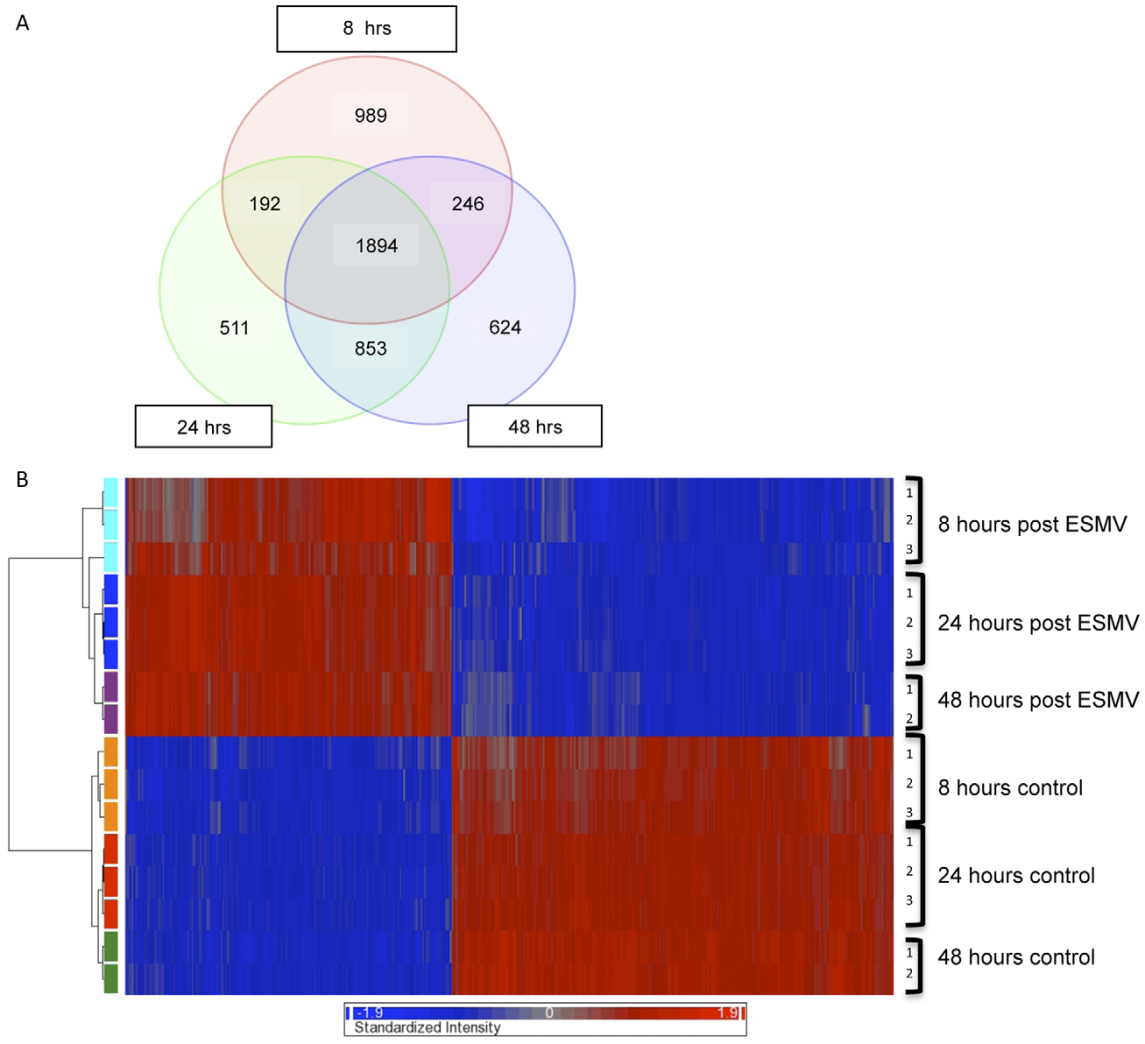
Microarray analysis of gene expression changes in Müller cells post-ESMV treatment. (A) Venn diagram of gene expression changes in ESMV-treated vs. control Müller cells at 8, 24, and 48 hours post ESMV exposure. The expression of 1894 genes was altered by ESMV treatment at all three time-points. There was significant overlap in differentially regulated genes between 8 and 24 hours (192) and 24 and 48 hours (853) post-treatment. The majority of gene expression changes occurred by 24 hours post-ESMV exposure. (B) Heat map representation of heirarchal clustering of 16 samples based on the 1894 probes found to be differentially regulated in the Müller cells post-ESMV treatment vs. control (p-value<0.001 and a minimum of 3-fold difference in expression). These transcripts were clustered with conventional Heat map analysis, with red representing up- and blue representing down-regulation; rows represent the samples and columns represent the genes.

1894 genes were differentially expressed at all 3 time points post-ESMV treatment, with 801 genes up- and 1093 genes down-regulated (Figure 4A). Tight clustering of genes in ESMV-treated vs. control Müller cells was observed, with treated cells sharing a similar gene expression profile over a wide range of genes (Figure 4B). More than 60% of the gene expression changes occurred by 8 hours post-treatment. 1444 genes were up- and 1878 genes were down-regulated at 8 hours, 1623 genes were up- and 1828 genes were down-regulated at 24 hours, and 1711 genes were up- and 1907 genes were down-regulated at 48 hours post-ESMV treatment. The majority of gene expression changes (95%) occurred by 24 hours, with only 624 genes unique to the 48-hour time point (Figure 4A).

**Figure 5:**
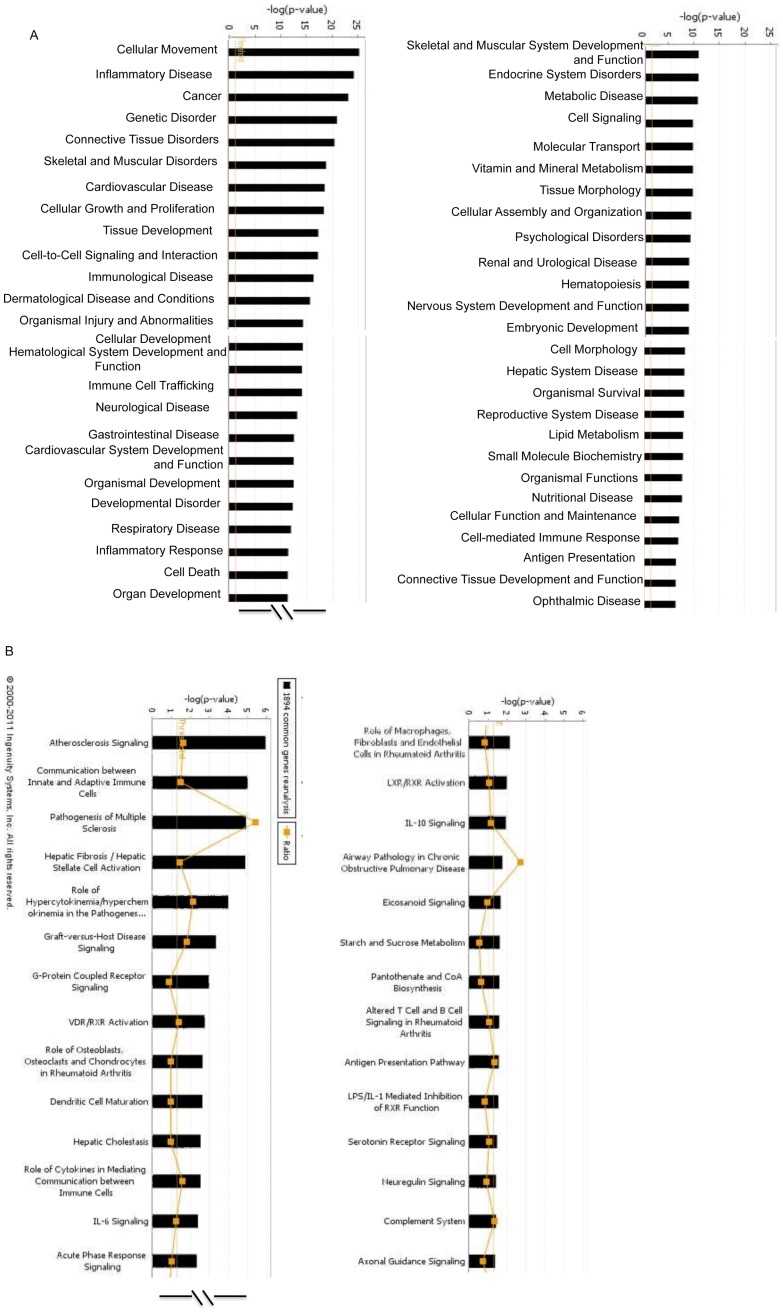
Ingenuity pathway analysis of 1894 genes differentially regulated at all tested time points between ESMV-treated and control Müller cells at p<0.001 level and fold change≥3. In this analysis, genes were tested for significant association in specific cell functional (A) or canonical cell signaling pathways (B) versus random change association in a total curated database of gene interactions of over 23,900 human, rat and mouse genes by right-tailed Fisher's exact test (Ingenuity Systems). A. Most significant biological functional groups. B. Most significant canonical pathways. Significance was assessed by testing the number of genes that were altered by ESMV treatments in a specific pathway versus total number of genes in this database for that pathway. The orange line indicates the threshold for a significant association, the –log(0.05).

Gene ontology (GO) analysis revealed that many of the genes differentially regulated at 24 and 48 hours post-treatment belonged to the transcription factor families, genes involved in retinogenesis, organ and organismal development, and genes encoding cell-cell signaling molecules, receptors involved in morphogenesis, multiple cytokines and immune response genes. Grouped by functional category (Figure 5A), ESMV-treated Müller cells differentially expressed multiple genes involved in cellular movement and extracellular matrix composition, inflammation, cellular growth and proliferation, tissue response to injury, molecular transport, energy metabolism, embryonic development, cell survival, DNA replication, and genes involved in ophthalmic diseases (Table S1). Many of the 1894 genes differentially expressed in ESMV-treated Müller cells at all time points have been linked, among others, to the following canonical signaling pathways: communication between innate and adaptive immune cells, G-protein coupled receptor signaling, vitamin D receptor and retinoic acid X receptor activation (important in the development of neural retina [33]), IL6 signaling (known retino-protective pathway [34]), neuregulin signaling (a pathway known to play a role in promoting retinal neuron survival and neurite outgrowth in developing retina [35]) and axonal guidance (Figure 5B).

Among the up-regulated genes were pluripotency genes *Oct4, Lin28, Klf4,* and *LIF*, early retinal genes *Bmp7, Olig2, FoxN4, Dll1, Pax6*, and *Rax*, genes with known retinal protective properties (*IL6*, *CSF2*) and inducers of retinal regeneration (*Fgf2, IGF2, GDNF*), as well as multiple extracellular matrix modifying molecules, such as the gene for Matrix metalloproteinase 3 (*MMP3*), that are known to create permissive environment for tissue remodeling. Among the down-regulated genes were those promoting differentiation, such as *DNMT3a* and *GATA4*, inhibitory extracellular matrix components such as *Aggrecan, Heparan Sulfate*, and *Tenascin*, and inhibitory scar tissue components such as *GFAP* and *chondroitin sulfate proteoglycans*. Expression changes in these genes were more pronounced at 24 and 48 hours post-treatment. While the expression of *c-Myc*, a well-characterized pluripotency-inducing factor [36], was detected in Müller cells, it remained unchanged throughout the course of ESMV treatments. Interestingly, *Hes1, Notch 1, Notch2*, and *NeuroD1*, genes believed to regulate cell cycle re-entry, de-differentiation, and activation of retinal stem cell phenotype in Müller cells [18], were most up-regulated at 8 hours post-ESMV treatment, with levels remaining increased over baseline, but declining at other time points. The expression of *EGFR*, a gene involved in driving retinal progenitors towards Müller glial fate during retinogenesis [20] was down-regulated at all three time points. The observed changes in Müller cells' transcriptome induced by ESMV treatments suggest a shift towards a more de-differentiated state, possibly through the activation of the proliferative and regenerative programs of these cells.

Additionally, microarray data analysis revealed the up-regulation of several genes encoding markers of various retinal lineages in Müller cells exposed to ESMVs, including those for calbindin 1, a marker of horizontal and amacrine retinal neurons, syntaxin 1a, a marker of amacrine cells, and rhodopsin, a marker of rod photoreceptors. The expression of calbindin 1 was highest 48 hours post-ESMV, while the expression of rhodopsin and syntaxin 1a was increased at all tested time points. These findings suggest that subsets of de-differentiating Müller cells may trans-differentiate into cells of other retinal lineages.

### Confirmation of ESMV-induced gene expression changes

**Figure 6:**
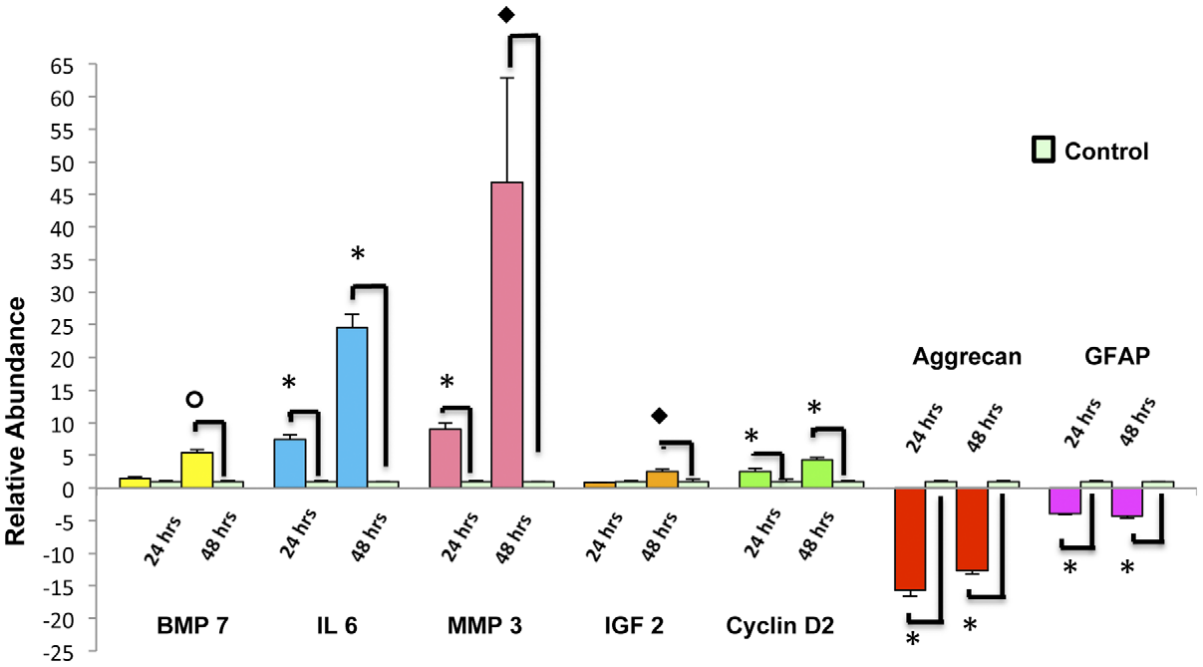
qRT-PCR analysis confirmed gene expression changes of microarray-identified genes in Müller cells at 24 and 48 hours post-ESMV treatment compared to untreated controls. Each bar represents the relative abundance of the genes tested in ESMV-treated vs. untreated Müller cells. Error bars represent S.E.M.; all samples were tested in at least three biological replicates, run in triplicates; Student's t-test was used to assess significant differences between experimental and control groups. *, p<0.00001; ⧫, p<0.0005; and **o**, p<0.001. *Gapdh* was used for normalization.

We verified with qRT-PCR of RNA from Müller cells subjected to ESMV treatment for 24 and 48 hours the expression profiles of a subset of genes involved in the processes of de-differentiation (*Cyclin D2, BMP7*), retinal protection (*IL6, IGF2*), repair and tissue remodeling (*MMP3*), as well as genes involved in scar formation (*GFAP*) and inhibition of ECM components (*Aggrecan*), all of which were significantly altered in the ESMV-treated group on the microarrays. These time points were chosen because the majority of changes in the expression of genes involved in retinal protective and regenerative processes were observed within 24 and 48 hours. All tested genes demonstrated the same pattern of regulation as observed in microarrays (Figure 6). In particular *MMP3* mRNA, which encodes a matrix metalloproteinase that up-regulates in newt adult organ repair, including retina, and facilitates the integration into the retina of transplanted photoreceptors when present at elevated levels [37], [38], was found strongly up-regulated in microarrays and qRT-PCR experiments, as was *IL6* mRNA; its expressed interleukin has been demonstrated to have a protective effect on inner retina neurons [39]. On the other hand, the *Aggrecan* gene, which encodes a chondroitin sulfate proteoglycan required for normal glial cell differentiation and development [40] was among the genes down-regulated in microarrays and qRT-PCR studies, as was *GFAP*, a gene that when deleted from the mouse genome improves retinal transplant integration [37].

### Genome-wide analysis of miRNA expression changes induced in Müller cells by ESMV exposure

**Figure 7:**
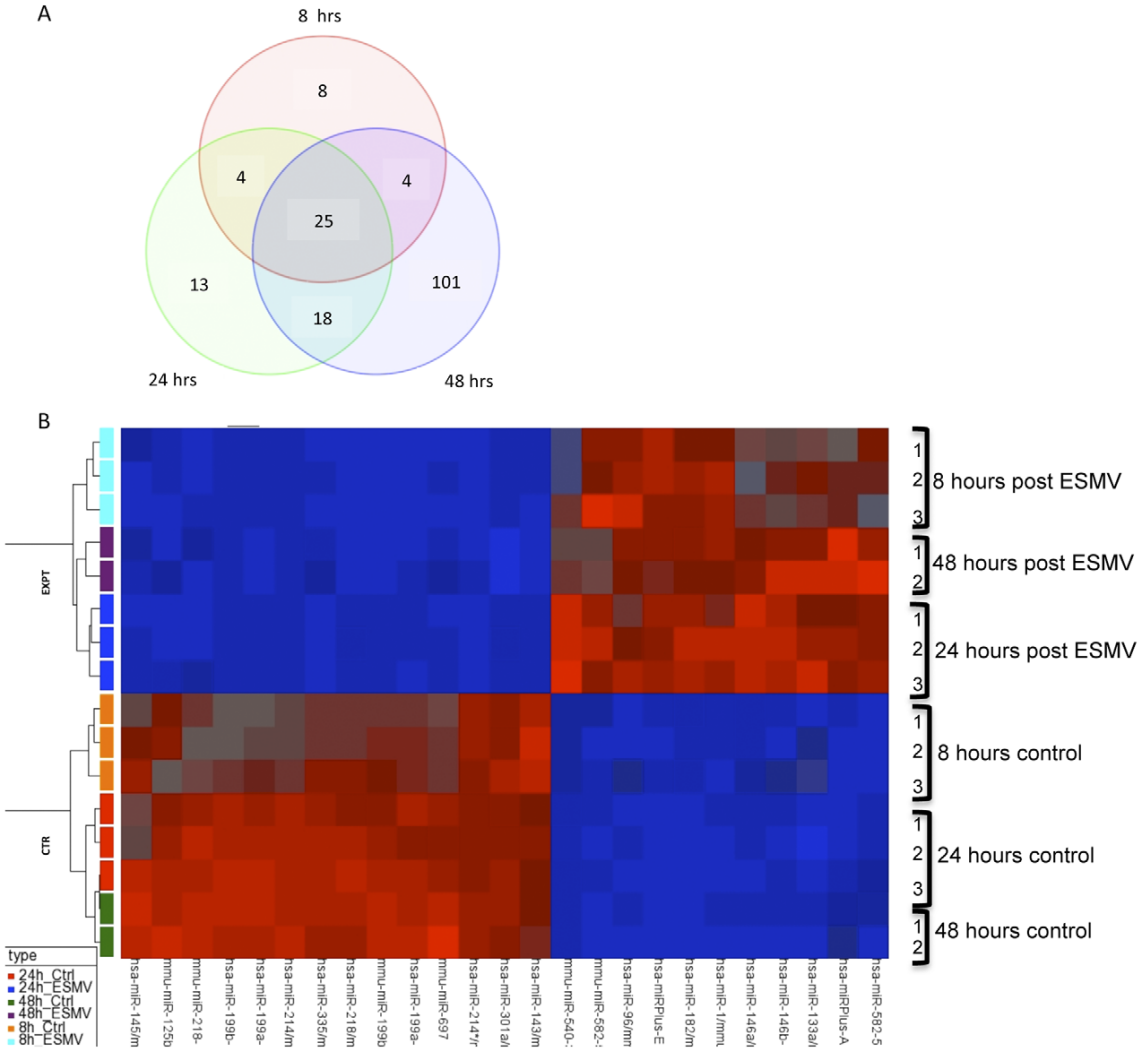
Microarray analysis of miRNA expression changes in Müller cells post-ESMV treatment. (A) Venn diagram of miRNA expression changes in ESMV-treated vs. control Müller cells at 8, 24, and 48 hours post-ESMV treatment. The expression of 25 miRNAs was altered at all three time points tested. The majority of the miRNA expression changes occurred at 48 hours post-ESMV, progressively increasing from 41 miRNAs at 8 hours, to 60 miRNAs at 24 hours, and 148 at 48 hours. (B) Heat map representation of hierarchal clustering of 16 samples based on 25 miRNA probes differentially regulated in ESMV-treated vs. control Müller cells at all times tested. (p<0.05, minimum 3-fold difference in expression). Each row represents a single sample, and each column-a single miRNA. The red or blue color represents relatively high or low expression, respectively. The two main groups of the dendrogram, labeled EXPT and CTR, separate treated vs. control groups of Müller cells.

In addition to their increasing recognition as important regulators of mammalian gene expression, cell cycle regulation, and cell fate determination, recent evidence implies that miRNAs play a role in retinogenesis, regulating retinal progenitor cells' progression from early to late stages and their differentiation towards various retinal cell lineages [41]. We hypothesized that miRNAs delivered to Müller cells by ESMVs may alter the miRNA and mRNA expression profiles of Müller cells and shift these cells towards a de-differentiated state. We used a portion of the total RNA used in the microarray studies described above to examine the global miRNA transcription changes in Müller cells post-ESMV treatment in parallel with global mRNA expression changes. Exiqon mercury LNA microRNA Arrays, which include 927/648/351 human/mouse/rat miRNAs as well as 438 miRPlus miRNAs were used in our studies. The obtained raw miRNA data were normalized using a combination of housekeeping miRNAs and invariant miRNAs. The threshold to select miRNAs with significantly altered expression was set at ≥3 fold change and FDR-corrected p<0.05. 720 miRNAs were detected in Müller cells. Overall, the expression of 173 miRNAs was altered by ESMV treatment; 25 of these miRNAs were differentially expressed at all three time points tested, with 11 up- and 14 down-regulated (Figure 7). 25 miRNAs were up- and 16 down-regulated at 8 hours, 32 miRNAs were up- and 28 down-regulated at 24 hours, and 87 miRNAs were up- and 61 were down-regulated at 48 hours post-ESMV treatment (Figure 7A). The majority of alterations in miRNA expression occurred by 48 hours post-ESMV exposure.

While the exact functions of miRNAs are largely unknown, processes involving particular miRNAs and miRNA-specific targets are gaining recognition. Several of the miRNAs previously noted to be highly expressed in developing retina were up-regulated in ESMV-treated Müller cells, including miR-1, miR-96, miR-182 and miR-183. miRNAs belonging to the 290 cluster (mir-291b-5p, -292, -294, and -295), the miRNA cluster involved in the maintenance of ESC pluripotency [42], were up-regulated and remained increased over 48 hours post-ESMV exposure. Concurrently, the expression of miR-let-7b and miR-let-7c, belonging to the miR-let-7 cluster known to inhibit cell cycle progression and promote cell differentiation [43], decreased post-ESMV treatment. miR-7, which represses the expression of Yan protein and promotes photoreceptor differentiation [44], as well as miR-125-2b, highly abundant in adult retina [45], were down-regulated over 48 hours post-ESMV treatment. Among the miRNAs strongly up-regulated at all three time points tested were miR-133a (increased 30-fold) and miR-146a (increased 37-fold), the miRNAs known to promote cell proliferation and inhibit differentiation of skeletal myoblasts and myogenic stem cells, respectively, the latter acting via the Notch signaling pathway, the same pathway known to regulate retinal progenitor differentiation [22]. Among the miRNAs strongly down-regulated at all tested times were miR-199b-5p (decreased 70-fold), miR-214 (decreased 37-fold), and miR-143 (decreased 13-fold), known to promote differentiation of ESCs, neuroblasts, and smooth muscle progenitors, respectively [46], [47]. The observed profile of miRNA expression changes in Müller cells post-ESMV exposure was suggestive of de-differentiation, consistent with that observed for mRNA expression changes.

### Confirmation of ESMV-induced miRNA expression changes

**Figure 8:**
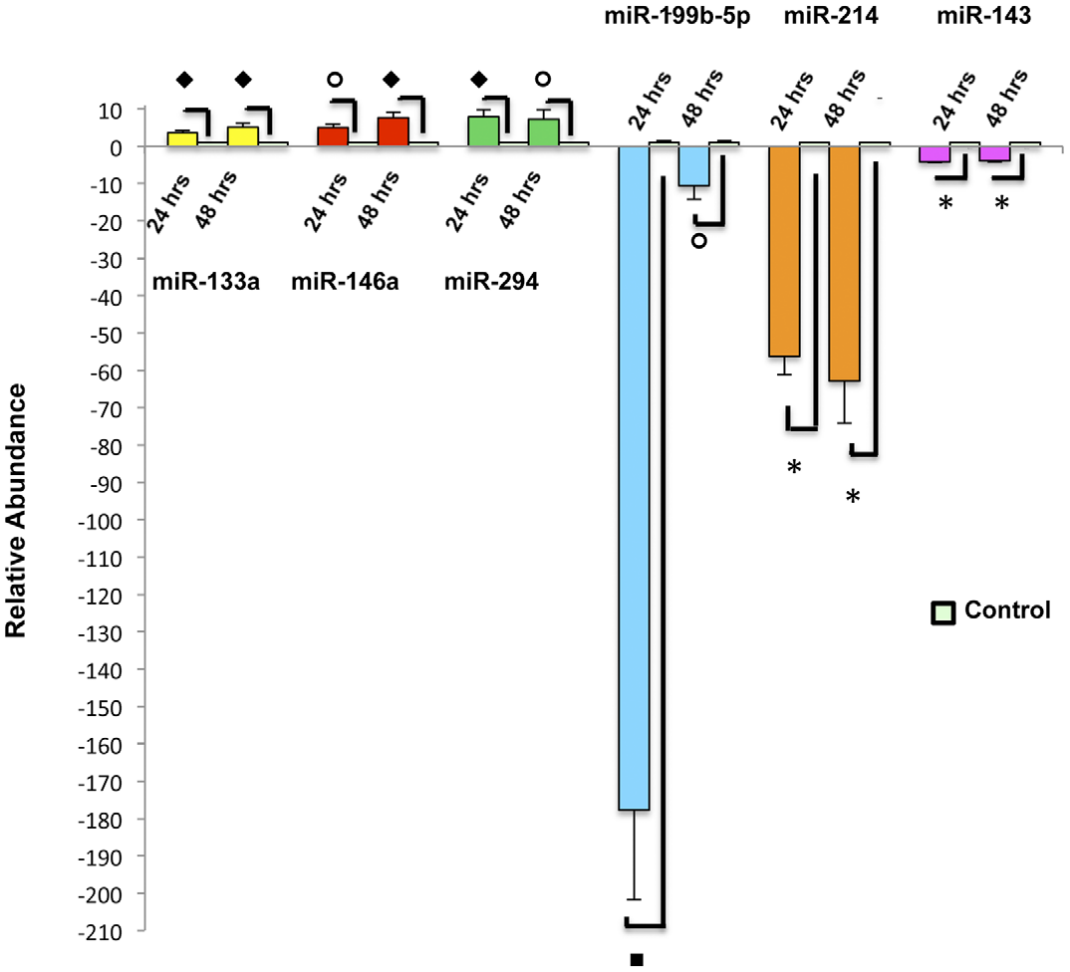
qRT-PCR analysis of select miRNAs involved maintenance of pluripotency (294), de-differentiation (133, 146), cell fate determination and differentiation (199, 214, 143), noted to be differentially expressed on in ESMV-treated vs. control Müller cells on miRNA microarrays. Each bar represents the relative abundance of the miRNAs tested in ESMV-treated Müller cells vs. untreated control cells. Error bars represent SEM; all samples were tested in at least three biological replicates, run in triplicates; Student's t-test was used to assess significant differences between experimental and control groups. *, p<0.00001; ⧫, p<0.0003; ▪, p<0.001 and o, p<0.005. U6 snRNA was used for normalization.

Several miRNAs involved in maintenance of pluripotency (miR-294, -146a, -133a) and differentiation (miR-199b-5p, -214, -143) were selected for validation of the microarray results. Total RNA samples from Müller cells at 24 and 48 hours post-ESMV treatment (the time points corresponding to the majority of miRNA expression changes) and from untreated control cells were subjected to qRT-PCR using Taqman® miRNA Assays that included stem-loop RT primers specific for each miRNA. qRT-PCR results confirmed the pattern of expression observed by the microarray screening for all the miRNAs tested (Figure 8).

### Immunocytochemical analysis of ESMV-induced Müller cell transdifferentiation

**Figure 9:**
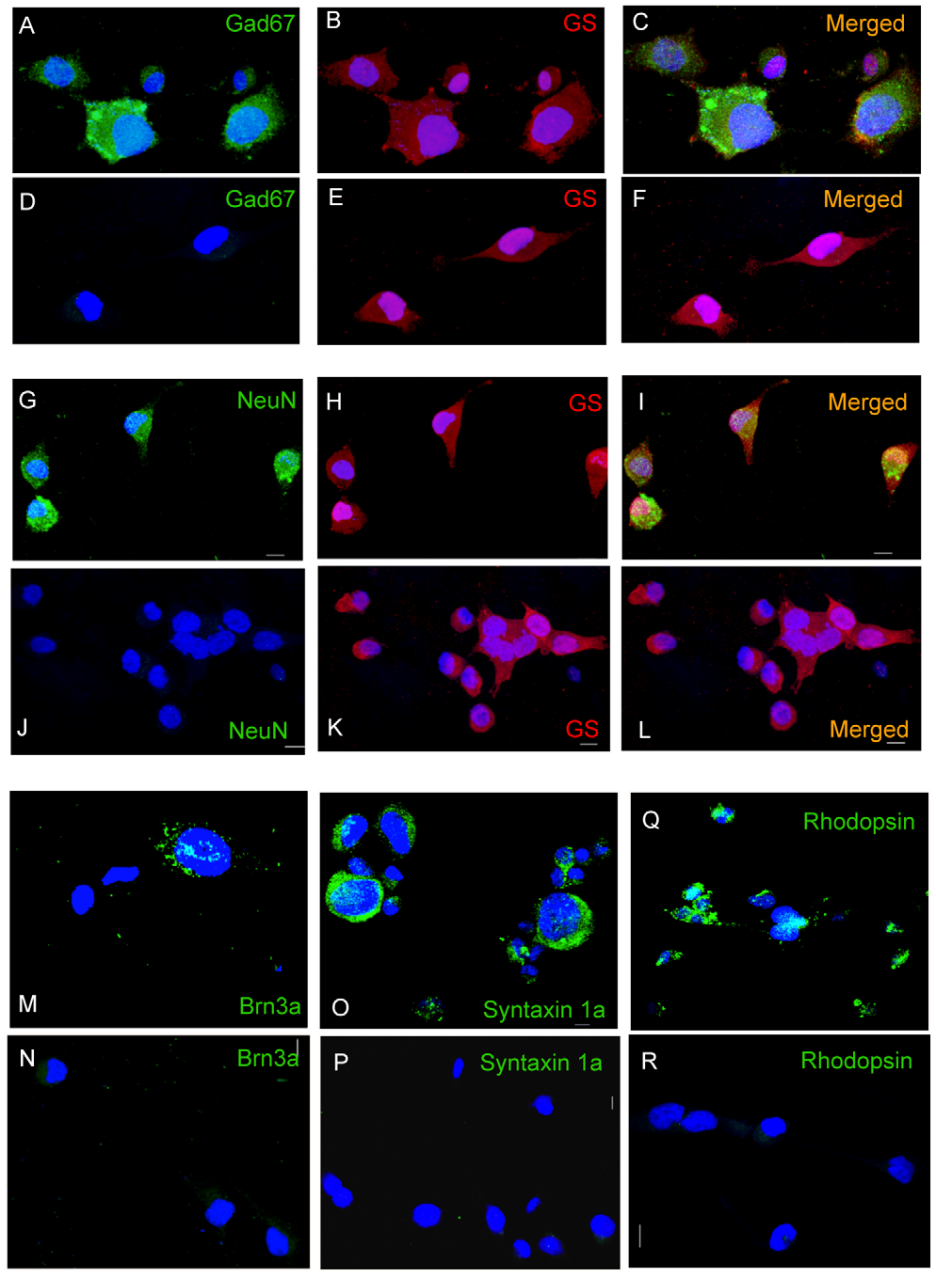
Confocal photomicrographs of ESMV-treated and control Müller cells immunostained for markers of various retinal lineages. In A–L, cells were double stained with Gad67 (amacrine & horizontal cells; green) or NeuN (amacrine and ganglion cells; green) and the marker of Müller cells, Glutamine Synthetase (red). (A–C): Gad67-stained ESMV-treated Müller cells. (D–F) Gad67-stained control Müller cells. (G–I) NeuN-stained ESMV-treated Müller cells. (J–L) NeuN-stained control Müller cells. The third panel of each row shows the merged first two images. (M) ESMV-treated and (N) control Müller cells, stained for Brn3a (green), a marker of retinal ganglion cells. (O) ESMV-treated and (P) control Müller cells stained for Syntaxin 1a (green), a marker of amacrine cells. (Q) ESMV-treated and (R) control Müller cells stained for rhodopsin (green), a marker of rod photoreceptors. Cell nuclei were labeled with 4′6-diamidino-2-phenylindole (DAPI, blue). Scale bar 10 μm for all panels. Images show z-axis projections of 15×1 μm in all channels.

To further characterize the morphologically heterogeneous cell population observed in the cultures of Müller cells treated with ESMVs and validate the microarray data, we investigated the expression of markers of various retinal cell lineages in ESMV-treated Müller cells compared to untreated control cells by immunocytochemistry. In addition to the Müller cell marker, glutamine synthetase (GS), we observed immunoreactivity to Gad67, a marker of amacrine and horizontal cells (Figure 9, A and C), NeuN, a marker of amacrine and retinal ganglion cells (Figure 9, G and I), Brn3a, a marker of retinal ganglion cells (Figure 9M), and syntaxin 1a, a marker of amacrine cells (Figure 9O) in small populations of ESMV-treated Müller cells. None of these markers were present in the untreated control cultures. Interestingly, immunoreactivity to rhodopsin, a marker of rod photoreceptors, was seen primarily localized in cytoplasmic granules in a very small number of treated cells (Figure 9Q). No immunoreactivity to Parvalbumin, a bipolar and horizontal cell marker, or Vesicular Glutamate Transporter 1, a marker for bipolar and photoreceptor cell terminals, was found. No staining was observed when primary antibodies were omitted. Our data suggest that ESMV treatment induces transdifferentiation of Müller cells into cells of retinal neural lineage, mainly towards amacrine and retinal ganglion cells, but not horizontal or bipolar cells. The very limited expression of rhodopsin post-ESMV exposure also suggests that ESMV treatment may induce at least a partial activation of genes of photoreceptor lineage.

## Discussion

ESMVs selectively transfer mRNA, microRNA, and proteins from their parent cells to recipient cells, likely altering gene expression, epigenetic state, and ultimately, cell fate of their recipients. We studied the effects of ESMVs on the morphology, mRNA and miRNA expression of retinal Müller cells. These cells have been shown to re-enter the cell cycle and regenerate all retinal layers in lower vertebrates [48], [49] and to exhibit progenitor-like gene expression and activity in mammalian retinas, generating retinal neurons, including photoreceptors, *in vitro* [18], [50], [51] as well as *in vivo* after retinal injury, when treated with a growth factor regimen [16], [17], [22], [23]. However, this response has been limited, likely due to the inhibitory effects of the microenvironment of the adult mammalian retina and, possibly, insufficient pro-regenerative stimulation. This study is the first to demonstrate the morphological, mRNA and miRNA expression changes towards a more de-differentiated phenotype induced by ESMV treatments in cultured Müller cells of the adult human retina. Moreover, we also observed changes in the expression of ECM building blocks, inhibitory scar tissue components, and ECM-modifying enzymes, suggestive of a shift in cellular microenvironment towards a more permissive state for tissue regeneration. Thus, our results possibly indicate that ESMVs may modify cell fate as well as cellular microenvironment toward a more de-differentiated, pro-regenerative state.

We demonstrated before [6] the transfer by ESMVs of mRNAs encoding transcription factors important for maintenance of ESC pluripotency, *Oct4* and *Sox-2,* to fibroblasts and other ESCs and now show that ESMVs also transfer these mRNAs to human Müller cells, Moreover, there was an up-regulation of endogenous *Oct4* mRNA of Müller cells following ESMV exposure. The greatest levels of *Oct4* mRNA, both transferred and endogenous, were observed at 8 hours post-ESMV, but both persisted for 48 hours, suggesting that *Oct4* mRNA was utilized in these cells over time. The lack of *Nanog* mRNA transfer, despite its abundance in ESMVs, suggests that there exists a selection mechanism to direct the genetic transfer, or that only a subset of mRNAs transferred are retained by the recipient cells, while the rest are rapidly degraded. ESMVs transferred to Müller cells miRNAs 292 and 295, the ESC cell cycle-regulating miRNAs involved in maintenance of ESC pluripotency, which, when introduced into somatic cells along with the *Klf4, Oct4* and *Sox2* genes promote the somatic cell reprogramming towards pluripotency [42]. The persistence of these transferred miRNAs for 48 hours suggests that they are utilized by the Müller cells, and likely facilitate the induction of embryonic pluripotency-promoting transcription factors, such as *Oct4* [28]. Our observation of the transfer of pluripotency-inducing mRNAs and miRNAs by ESMVs to Müller cells supports the hypothesis that ESMVs may serve the function of physiologic liposomes, delivering the de-differentiation signals to the quiescent progenitor cells. In light of the recent demonstrations of mesenchymal and bone marrow stem cells augmenting regeneration of cardiac and vascular tissue after ischemic injuries [52] and the observations of microvesicles derived from several adult stem cell populations augmenting the repair of renal, hepatic, and lung tissues, the microvesicle-mediated transfer of pluripotency-inducing mRNAs and miRNAs observed in our study offers an insight into the paracrine mechanim by which stem cells may be able to augment tissues' endogenous regenerative capacity, bypassing the need for tissue engraftment.

Pax6 is among the earliest transcription factors to appear in the retina and it is involved in the entire process of retinogenesis [26]; it is a marker of neurogenic radial glia in the adult CNS [53] and of retinal progenitor cells [25]. A subset of adult Müller cells has been demonstrated to express low levels of Pax6, corroborating the recognition of these cells as adult retinal progenitors [17]. We observed an increase in *Pax6* mRNA levels in our Müller cell samples beginning at 8 hours post-ESMV exposure, progressively increasing to more than 5-fold 48 hours after treatment. Similarly, the expression level of the *Rax* mRNA – also one of the first to appear homeobox transcription factors that defines the eye field during early development and is involved in determination of photoreceptor cell fate – was strongly (>100-fold) induced 8 hours post-ESMV treatment, with high, albeit declining levels remaining 40 hours later. Both Pax6 and Rax direct retinal cell differentiation during embryogenesis [54] and during the derivation of retinal cell types from ESCs and retinal progenitor cells [55], [56]. The observed expression changes suggest that ESMV-treated Müller cells shift towards a more de-differentiated state, assuming an early retinal progenitor phenotype and possibly reactivating the developmental program involved in retinogenesis. Re-activation of the quiescent resident progenitors in the retina opens the possibility of retinal repopulation without the need for utilization of ESC-derived retinal layers. Since retinal resident progenitors are epigenetically programmed to assume the retinal lineage, the transdifferentiation of these cells will offer a safer, less invasive alternative to cell transplantation.

ESMV treatment induced a robust global gene expression change in Müller cells, activating a distinct transcriptome, which differed markedly from that of untreated cells. Using stringent statistical parameters to select a set of genes that changed expression significantly post-ESMV treatment, we identified 1894 distinct genes common to all of the post-treatment groups (8 hours, 24 hours, and 48 hours). The analysis of the post-treatment transcriptome showed enrichment in the embryonic pro-pluripotency genes, early retinal genes, retinoprotective genes, and genes known to induce retinal progenitor proliferation and regeneration, and depletion of pro-differentiation genes and glial scar markers, consistent with our observations of Müller cells' morphological changes towards a more de-differentiated phenotype and the results of our initial gene expression studies. Among the differentially regulated genes were also those coding for ECM components and ECM-modifying molecules, their changes reflecting a shift from scar formation to a tissue remodeling profile. These findings indicate that ESMVs are able to alter the gene expression of the target cells as well as their cellular microenvironment.

ESMV exposed Müller cells up-regulate several of the factors required for generating induced pluripotent cells, including *Oct4, Klf4*, and *Lin28* [36], [49], [57] as well as the retinal progenitor transcriptome. The expression of many of these progenitor genes increased at different times post-ESMV exposure. For example, *Vsx-1* and *Vsx-2*, homeobox genes first identified in the peripheral margin of the adult goldfish retina, were most up-regulated 24 hours post-treatment and their levels declined by 48 hours. Interestingly, the expression of these genes is restricted to the mitotically active adult progenitor cells and declines as the cells become post- mitotic and begin to differentiate [58]. The expression of archaete-scute homolog 1 (*Ascl1*), a gene that is up-regulated early in the process of regeneration in fish retina and that when inhibited prevents the regeneration from Müller cells [59], was also increased 24 hours post-ESMV. Delta-like 1 (*Dll1*), a gene that plays a key role in maintaining a pool of progenitors during the period of retinogenesis [60], was most up-regulated 8 hours after treatment, with levels steadily declining thereafter. The expression of *FoxN4*, a transcription factor that appears at the earliest stages of retinogenesis and is necessary for induction of progenitor cell differentiation towards amacrine and horizontal cells as well as for the proper axonal guidance of retinal ganglion cells [61], was increased early post-treatment and remained elevated 48 hours later. *Prox1*, which encodes a transcription factor expressed in the adult retinal progenitors of teleost fish and in rod photoreceptor precursors [62], a likely downstream target of *FoxN4* [63], was increased throughout the ESMV treatments, exhibiting high levels 48 hours post-ESMV. *Neurog2*, a gene important in directing retinal progenitors toward neuronal fate, was up-regulated at 24 hours [64]. As with the eye homeobox transcription factors Pax6 and Rax, expression of the gene encoding Bone Morphogenic Protein 7 (*BMP7*) markedly increased at all post-treatment times. This is a transcription factor critical for many steps in the progressive development of the eye, including patterning of the eye field as well as the formation of retinal pigmented epithelium and retinal ganglion cells [65]. The Notch pathway plays a pivotal role in maintaining the retinal progenitor pool and controls the activation of the progenitor program in Müller cells [18], [23], [66]. Several of the Notch pathway components, including *Notch1, Dll1*, and *Hes1*, were up-regulated in our samples post-ESMV treatment. Taken together, these findings strongly suggest that ESMV treatments induce de-differentiation of Müller cells and activation in these cells of the retinal progenitor phenotype. Moreover, the activation of early and late retinal progenitor genes at different times post-ESMV treatment and the up-regulation of the transcription factors that direct retinal progenitor differentiation towards multiple retinal lineages open the possibility that Müller cells, once induced to enter the retinal progenitor state by ESMVs, activate a retinogenic program. Indeed, ESMV treatment induced a small fraction of Müller cells to up-regulate the genes for several markers of retinal amacrine, horizontal, and ganglion cells as well as rhodopsin, a marker of rod photoreceptors, suggesting transdifferentiation along several retinal lineages.

Immunocytochemical analysis of ESMV-treated Müller cells revealed the presence of cells expressing the marker proteins for amacrine lineage Gad67 and Syntaxin 1a, cells expressing Brn3a and NeuroN, markers of retinal ganglion cells, and cells expressing rhodopsin, a marker of rod photoreceptors. We did not find any evidence of cells differentiating along the lines of bipolar or horizontal interneurons. It has been previously demonstrated that Müller glial cells are the source of pan-retinal, including neuronal, regeneration in fish [17], [59], [67], and to a lesser extent, in post-hatch chicks, with spontaneous differentiation into amacrine and bipolar neurons after injury in the latter [22]. Although mammalian Müller cells do not spontaneously re-enter the cell cycle after retinal injury, several groups have reported successful induction of Müller cells to enter the cell cycle and generate new amacrine [23] as well as bipolar and horizontal cells and rod photoreceptors [16] after treatment of injured retinas with various combinations of growth factor regimens, including fibroblast growth factor, epidermal growth factor, retinoic acid, and insulin. Here we demonstrate that ESMVs induce human Müller glia to de-differentiate, turn on an early retinogenic program, and differentiate towards cells of amacrine, ganglion cell, and rod photoreceptor lineage *in vitro*. Future studies will determine whether ESMVs can efficiently activate retinal resident progenitor Müller cells *in vivo* to augment retinal regeneration after injury. Since the cellular microenvironment plays a major role in cell fate determination and differentiation in developmental and regenerative processes, it is possible that, once activated, the resident Müller cells may differentiate into cells of other retinal lineages *in vivo*, guided by environmental cues.

Another pattern of gene expression changes in Müller cells post-ESMV exposure that emerged from our global gene expression analysis was the increase in genes that play a retinoprotective role and genes that stimulate retinal repair after injury. ESMV treatment induced the activation of genes involved in multiple cytokine pathways and immune response genes, including interleukin signaling, that have been studied extensively as therapeutic agents in the preservation of retinal ganglion cells [34]. The expression of IL6, an interleukin found to prevent photoreceptors from degeneration after retinal detachment [39] as well as retinal ganglion cell apoptosis in the setting of increased intraocular pressure [34], was strongly increased (9, 24, and 18-fold at 8, 24, and 48 hours, respectively) post-ESMV treatment. Glial- derived neurotrophic factor (GDNF), a growth factor shown to exert long-term neuroprotection when secreted from Müller cells in a rat model of retinitis pigmentosa [68] and increase long-term ganglion cell survival in glaucoma [69] was also strongly up-regulated at all times post-ESMV administration. The expression of neuregulins 1 and 2, known to promote survival and neurite extension from retinal neurons during retinal development [35], and FGF2, a growth factor demonstrated to slow down photoreceptor degeneration in retinitis pigmentosa [70] and promote retinal regeneration from progenitor cells in Xenopus [71] were also increased significantly at all times post-ESMV exposure. These results suggest that ESMVs activate a retino-protective program in Müller cells along with activating the regeneration program.

Together with the genes that dictate Müller cell fate and behavior, ESMV treatment induced gene expression changes in the genes coding for ECM components and ESC modification molecules. Of note, multiple matrix metalloproteinases (MMP) were strongly up-regulated at all time points, the most dramatic up-regulation of more than 200-fold observed in the *MMP3* transcript. MMPs are a group of enzymes that degrade inhibitory ECM components, such as basement membrane molecules, creating a permissive environment for tissue regeneration and integration of transplanted cells in the retina [38]. Concurrently, molecules encoding the ECM components shown to inhibit neurite outgrowth and retinal regeneration, such as *Aggrecan, Heparan Sulfate, Versican*, and *Decorin* [72], as well as inhibitory glial scar components, such as *GFAP* and *Tenascin C* [37], were strongly down-regulated. Thus, ESMV treatment directs the changes in cellular microenvironment towards a more permissive state for tissue regeneration and rewiring of neural circuitry within the retina. Elements of extracellular microenvironment normally present within the retina, subretinal space, and adjacent tissues, such as the retinal pigment epithelium and Bruch's membrane, have a significant effect on cell fate determination of retinal progenitors [73]. Activation of the resident progenitors within the damaged retina to de-differentiate and re-enter the cell cycle may lead to regeneration of those cells lost secondary to retinal damage in each particular disease or injury.

miRNAs play important roles during embryonic development as well as in cell fate determination and maintenance of the differentiated state [13], [27], [42], [74], [75]. Distinct patterns of miRNA expression have been observed at different stages of retinal development [41], [45]. Our results suggest that exposure of human Müller cells to ESMVs profoundly alters their miRNA expression profile, since we observed expression of miRNAs transferred by the ESMVs as well as changes in their endogenous miRNAs. Thus, ESMVs seem to induce a distinct change in the epigenetic state of Müller cells. For example, several ESC miRNAs of the 290 cluster were transferred from ESMVs and persisted in Müller cells for at least 48 hours. The miRNA-290 cluster acts through multiple mechanisms to promote pluripotency state and oppose differentiation of ESCs and is down-regulated upon differentiation and undetectable in adult organs [28]. Moreover, several members of the miRNA-290 cluster have been shown to enhance the efficiency of generation of induced pluripotent stem cells from adult somatic cells [42]. The transfer of these miRNAs may play a role in the induction of the endogenous pluripotency genes in Müller cells, observed in our microarray analysis, supporting the shift of Müller cells towards a progenitor state. In addition, we observed the up-regulation of endogenous human miR-1, miR-96, miR-182, and miR-183, the appearance of which marked progression through early retinal development [45], supporting the notion that ESMV treatment shifts Müller cell differentiation towards an early retinal progenitor stage. The concurrent down-regulation of the miR-let7 cluster, known to promote differentiation in most cells and de-differentiation when it is inhibited [76], miR-125, a highly abundant miRNA in adult retina [45], and miR-7, known to promote photoreceptor differentiation [44] is consistent with Müller cell de-differentiation under the influence of ESMVs. The majority of the miRNA expression changes occurred by 48 hours post-ESMV exposure, suggesting long-term alterations in the epigenetic state of Müller cells, and, effectively, in Müller cell fate.

Microvesicles have been demonstrated to augment functional regeneration of hepatic tissue in partially hepatectomized rats [9], induce bone marrow cells to repopulate radiation-injured lung [10], and protect and repair acutely and chronically injured kidneys [12]. In light of these results and the observation that ESMVs induce de-differentiation and pluripotency of Müller cells after prolonged exposure, our data opens the possibility of employing ESMVs as therapeutic agents to induce and support the retina's endogenous regenerative capacity. Future *in vivo* studies of the effects of ESMVs on retinal repair will be needed to explore the mechanism of ESMV action on retinal tissues and their efficiency in the *in vivo* induction of retinal regeneration.

## Materials and Methods

### Ethics Statement

All experiments involving mice were carried out using protocols approved by the UCLA Animal Research Committee, and in accordance with the ARVO Statement for Use of Animals in Ophthalmic and Vision Research.

### Müller Cell Culture

The human Moorfield/Institute of Ophthalmology-Müller 1 (MIO-M1) cell line, initially derived from postmortem human neural retina, was established and characterized previously [77]. MIO-M1 cells were maintained as an adherent cell line in 175 cm^2^ tissue culture flasks for propagation, and in 6-well cell culture plates for ESMV treatment experiments, in DMEM medium containing 4500 mg/L glucose, sodium pyruvate and stabilized L-glutamine (GlutaMAX; Invitrogen, Grand Island, New York) with 10% vol/vol fetal bovine serum (filtered, heat inactivated; Gemini Bioproducts, Sacramento, CA) and penicillin/streptomycin (Invitrogen) in a humidified 37°C, 5% CO_2_ incubator. Upon reaching confluence, the cells were washed with phosphate-buffered saline (PBS), detached from the flasks with trypsin (Invitrogen), washed with complete cell culture medium, and split into fresh flasks. ESMV treatment experiments were started when Müller cells reached 60% confluence. ESMV-exposed and control Müller cells were examined after each treatment using the Leica DM IL LED microscope (Leica Microsystems, Wetzlar, Germany), and for the determination of cell number, images of 3-4 fields of view (acquired at 20X magnification for each well of a 6-well plate of treated and control cells) were obtained using a Leica DCF295 digital camera. Cells within each image were individually marked using Adobe Photoshop (Adobe Systems, San Jose, CA), and then counted. Cell number per field of view was obtained at each time point for treatment and control groups and ratios of treated/control cells were calculated. For morphology studies, Müller cells were fixed in 100% ethanol for 15 minutes and stained with Harris Hematoxylin and Eosin Y (Fisher Scientific, Pittsburgh, PA), dehydrated with serial ethanol washes, air dried and coverslipped with ProLong Gold antifade reagent (Invitrogen). The transmitted light differential interference contrast images were acquired using a Zeiss Axiovert 135M microscope with a Photometrics CoolSnap camera (Roper Scientific, Tucson, AZ).

### ESC culture and ESMV isolation

ESCs derived from the mouse strain SV129 were expanded under serum-free and feeder-free conditions in ESGRO Complete PLUS clonal grade medium supplemented with GSK3β inhibitor to suppress differentiation (Millipore, Billerica, MA). 3.5×10^6^ cells were plated on gelatin-coated T175 cm^2^ culture flasks. ESCs were cultured in a humidified 37°C, 5% CO_2_ incubator. The growth of ESCs was monitored microscopically and fresh culture medium was added daily and collected every 48 hours for ESMV isolation. ESCs were passaged using ESGRO Complete Accutase (Millipore) every 48–72 hours to maintain ESC colonies at 80% confluence in order to maximize ESMV yield while avoiding differentiation of ESCs. ESC colonies were visually inspected by microscopy on a daily basis for signs of differentiation and *Oct4, Sox2*, and *Nanog* mRNA expression was assayed by qRT-PCR [mouse specific primer pairs designed by PrimerQuest^SM^ (Integrated DNA Technology-DNAsite, San Diego, CA): *Oct4*: forward-GCCGGGCTGGGTGGATTCTC, reverse-ATTGGGGCGGTCGGCACAGG, *Nanog*: forward-TCCAGAAGAGGGCGTCAGAT, reverse-CTTTGGTCCCAGCATTCAGG, *Sox2*: forward-AACAATCGCGGCGGCCCGAGGAG, reverse-GCCTCGGCGTGCCGGCCCTGCG]. To isolate ESMVs, the supernatant was collected in 50 ml centrifuge tubes and spun at 3,500 g for 1 hour at 4°C to pellet debris and fragmented cells. The supernatant was carefully transferred to an ultracentrifuge tube and spun at 200,000 g for 3.5 hours in a Beckman Type 50.2Ti rotor at 4°C to pellet the ESMVs.

### ESMV RNA and miRNA content analysis

ESMVs' total RNA was isolated using the mirVana miRNA isolation kit, which retains small RNA species (Ambion, Austin, TX), treated with TURBO DNAse (Ambion) to remove DNA traces, and examined by RT-PCR for the presence of mouse *Oct4, Sox2, Nanog* (primer pairs above), and *Klf4*, *Lin28*, and mmu-miR-292-3p, -294, and -295 (Taqman® primers) transcripts.

### Treatment of Müller cells with ESMVs

Müller cells were plated on two 6-well cell culture plates at 1×10^6^ cells per well and allowed to reach 60% confluence prior to initiating ESMV treatments. For these, ESMVs pelleted by ultracentrifugation of media from 6 T175 cm^2^ flasks of mouse ESCs grown in serum free, feeder free conditions, were immediately resuspended in Müller cell medium and equal volume was added to each well of one of the 6-well plates with cultured Müller cells. This procedure was repeated every 48 hours for 9 consecutive treatments. Control Müller cells cultures in the other 6-well plate were subjected only to medium changes in place of ESMV treatments. To maintain 60% confluence, both treated and control cells were passaged as needed at the end of an ESMV treatment. ESMV-exposed and control Müller cells were examined after each treatment using the Leica DM IL LED microscope.

To evaluate the morphological changes induced by ESMVs at the completion of each treatment, Müller cells were fixed in 100% ethanol for 15 minutes and stained with Harris Hematoxylin and Eosin Y, dehydrated with serial ethanol washes, air dried and coverslipped with ProLong Gold antifade reagent. The transmitted light differential interference contrast images were acquired using the Zeiss Axiovert 135M microscope with a Photometrics CoolSnap camera. To compare the number of cells present in ESMV-exposed and control Müller cell cultures at the end of each treatment, images of 3–4 fields of view (acquired at 20X magnification for each well of the 6-well cell culture plates) were obtained using the Leica DCF295 digital camera. Cells within each image were individually marked using Adobe Photoshop, counted, and the treated/control cells ratios were calculated.

At the completion of ESMV treatments, the culture medium of ESMV-exposed Müller cells was aspirated; the cells were then washed 3 times with ample PBS to remove any residual ESMVs and collected for RNA isolation and gene expression studies.

### Müller cell RNA isolation and Real Time quantitative RT-PCR

For the initial analysis of gene expression changes in Müller cells post-ESMV treatment, total RNA was isolated using the mirVana^TM^ miRNA Isolation Kit (Ambion) from the collected cells and from control cells cultured and from control Müller cells cultured under three different conditions (Müller cells not exposed to ESMVs, Müller cells incubated with ESGRO medium components that remained after ultracentrifugation at 200,000 g for 3.5 hours, and Müller cells treated with the components of the conditioned medium of MEF cultures, after ultracentrifugation at 200,000 g for 3.5 hours). The RNA was quantified and quality assessed using a Nanodrop ND-1000 spectrophotometer (Thermo Scientific, Wilmington, DE) and treated with TURBO DNAse (Ambion) prior to further manipulation. RNA was converted to cDNA using SuperScript^TM^ III First-Strand Synthesis SuperMix for qRT-PCR (Invitrogen). To analyze embryonic gene transfer from mouse ESMVs to human Müller cells, mouse-specific primer pairs described above for *Oct4, Sox2*, and *Nanog* were used, and amplification was detected using Brilliant Sybr Green qPCR Master Mix (Stratagene, La Jolla, CA) in an Mx3000p qPCR instrument (Stratagene). All results were normalized to the human housekeeping gene glyceraldehyde 3-phosphate dehydrogenase (*Gapdh*), amplified using commercially available primers (IDT, Coralville, IA). The relative change in gene expression was determined using the 2^−ΔΔCt^ method of comparative quantification. To detect the induction of expression by ESMVs of endogenous embryonic and early retinal genes in human Müller cells, TaqMan® primers for the human *Oct4, Pax6*, and *Rax* genes and the Taqman® Gene Expression Assays protocol and reagents (Applied Biosystems, Carlsbad, CA) were used; TaqMan® *Gapdh* primers were used for normalization.

Comparative quantification of mmu-miR-292-3p and mmu-miR-295 with snRNA U6 (endogenous control) was performed in 3–6 biological samples, ran in parallel, using TaqMan® miRNA qRT-PCR assays and TaqMan® probes (Applied Biosystems) according to manufacturer's protocol, each primer ran in triplicate, and the 2^−ΔΔCt^ method was used to examine the fold-change of miRNA levels. The significance of these changes was assessed using Student's *t*-tests. The commercially available primer sequences for the TaqMan assays can be downloaded from the manufacturer's website (https://bioinfo.appliedbiosystems.com/genome/database/gene/expression.html).

### cDNA Microarray Gene Expression Studies

To study the transcriptome changes in Müller cells in response to ESMV treatment, control and ESMV-exposed Müller cells were collected at 8, 24, and 48 hours post-ESMV treatment (3 independent biological samples for 8 and 24 hours and 2 samples for 48 hours) and total RNA was isolated from each sample using the miRNeasy Mini kit for purification of total RNA, including miRNA (Qiagen, Valencia, CA, USA). The purified RNA samples were divided into two parts, one to be used for gene expression array hybridization and the other for miRNA array hybridization. For gene expression profiling, total RNA samples were reverse transcribed and each obtained cDNA, in triplicate, was hybridized to Agilent human 8X60k arrays. Hybridizations were performed by the UCLA Clinical Microarray Core facility following the standard Agilent Expression Analysis protocol. The acquisition of array images was carried out using the Agilent Scan Control and Feature Extraction 10.7 software. Subsequent raw data were analyzed using Partek genomics Suite 6.4. Global functional analyses, network analyses and canonical pathway analyses were performed using Ingenuity Pathway Analysis (Ingenuity® Systems, www.ingenuity.com). Additionally, to assess the relevance of the identified gene expression changes, searches for Gene Ontology-based functional groups overrepresented in the ESMV-treated cells and literature-based functional networks were performed using the Database for Annotation, Visualization and Integrated Discovery (DAVID; http://david.avcc.ncifcrf.gov/ref) online bioinformatics tool. Identification of enriched categories and biological processes among regulated genes was performed using the DAVID Functional Annotation Chart. Only categories with an enrichment score yielding a p-value <0.05 were considered significant.

### miRNA Microarray Expression Studies

One μg total RNA samples prepared as described above was labeled with Hy3^TM^ and the labeled miRNAs were hybridized to miRNA arrays by the UCLA Clinical Microarray Core facility. Exiqon miRCURY LNA miRNA arrays (microarrays v11), which include 927/648/351 human/mouse/rat miRNAs as well as 438 miRPlus miRNAs, were used according to the manufacturer's instructions. The miRNA array slides were scanned with an Axon GenePix 4100A scanner (Molecular Devices, Sunnyvale, CA) and processed with the GenePix Pro 6.0 software (Molecular Devices). The raw miRNA data were normalized using a combination of housekeeping miRNAs and invariant miRNAs. Statistically different miRNAs were selected using Partek genomic suite 6.4 with thresholds of ≥3-fold and FDR corrected p<0.05. Individual miRNAs were studied using the miRBase online database (http://www.mirbase.org/) and miRNA target prediction analysis was performed using TargetScan 6.0 software (http://www.targetscan.org/).

### Validation of transcript level changes

qRT-PCR analysis of independent samples of ESMV-treated and control Müller cells was carried out to validate the expression changes from the mRNA and miRNA microarray data. Since microarray analysis indicated that the majority of expression changes in the genes of interest take place 24 and 48 hours post-ESMV exposure, these time points were used for array validation. Total RNA was isolated using miRNeasy Mini kit (Qiagen) and subjected to on-column DNase digestion per protocol. For gene expression change validation, RNA was converted to cDNA as described above, and qPCR was carried out using the following TaqMan® primers, selected to span exon-exon junctions to eliminate potential genomic DNA amplification in the Expression Assay protocol (Applied Biosystems, https://products.appliedbiosystems.com/ab/en/US/adirect/ab?cmd= catNavigat2&catID = 601803): BMP7, IL6, MMP3, IGF2, Cyclin D2, Aggrecan, and GFAP, with Gapdh serving as the endogenous control. In each experiment, a sample without reverse transcriptase and a sample without template were included to demonstrate specificity and lack of DNA contamination.

For miRNA array validation the following TaqMan® miRNA assays (Applied Biosystems, link above) were used in accordance with manufacturer's protocol: hsa-miR-146a, hsa-miR133a, mmu-miR-294, hsa-miR-199-5p, hsa-miR-214*, hsa-miR-143, normalized against snRNA U6. Fold-change in gene expression was calculated using the 2^−ΔΔCt^ method for each mRNA and miRNA tested; 3 biological replicates were ran in parallel for each sample and each primer was ran in triplicate. Student's *t*-test was used to assess significance of gene expression change.

### Immunocytochemistry

To investigate which retinal cell-specific markers were expressed in ESMV-treated and control Müller cells, the Müller cells that had had 8 treatments with ESMVs were seeded on poly-D-lysine-coated glass coverslips placed in the 6-well culture plates, allowed to attach, and treated with ESMVs derived from 6 T175 flasks, as described previously. 24 hours later, ESMV-treated and control cells were rinsed in 0.1 M PBS and fixed for 30 minutes in 4% paraformaldehyde (Electron Microscopy Sciences, Hatfield, PA), then rinsed in 0.1 M PBS and blocked in 10% serum containing 1% bovine serum albumin (BSA) and 0.5% Triton X-100 for 1 hour at room temperature. Primary and secondary antibodies were diluted with PBS containing 3% serum, 1% BSA and 0.5% Triton X-100. Cells were incubated with the following primary antibodies, overnight, at 4°C: rabbit monoclonal anti-Brn3a (1∶500, Abcam, Cambridge, MA), mouse anti-Neuronal Nuclei (NeuN) monoclonal antibody (1∶500, Millipore) mouse anti-HPC1 (Syntaxin 1a) monoclonal antibody (1∶1000, Sigma, St Louis, MO), mouse anti-glutamic acid decarboxylase 67 (GAD67) monoclonal antibody (1∶1000, Millipore), mouse anti-1D4 (rhodopsin) monoclonal antibody (1∶10,000, Millipore), rabbit anti-GS polyclonal antibody (1∶1000, Sigma), mouse anti-parvalbumin monoclonal antibody (1∶1000, Swant, Marly, Switzerland) and guinea pig anti-vesicular glutamate transporter 2 (vGluT2) (1∶10,000, Millipore). ESMV-treated and control cells were then incubated for 1 to 2 hours at room temperature with the appropriate secondary antibodies conjugated to AlexaFluor488, AlexaFluor568, or AlexaFluor594 (Molecular Probes, Eugene, OR) and diluted 1∶1000. Coverslips were mounted on slides using ProLong® Gold Antifade Reagent containing the nuclear counterstain DAPI (4,6-diamidino-2-phenylindole; Invitrogen), allowed to dry, and images were obtained with an Olympus FluoView FV1000 confocal laser scanning microscope using Olympus FluoView software for capture and processing (Olympus America, Center Valley, PA).

## Supporting Information

Table S1 **Gene Ontology (GO) analysis of the 1894 genes that were differentially expressed in ESMV-treated vs. control Müller cells at all three time points tested by microarray analysis, grouped by functional category.**
(DOC)
