# Supplementary material for: Embryonic Stem Cell-Derived Microvesicles Induce Gene Expression Changes in Müller Cells of the Retina
Source: PLoS One. 2012 Nov 30;7(11):e50417. doi: 10.1371/journal.pone.0050417 (PMC3511553; doi:10.1371/journal.pone.0050417)
Supplement: Table S1 — Gene Ontology (GO) analysis of the 1894 genes that were differentially expressed in ESMV-treated vs. control Müller cells at all three time points tested by microarray analysis, grouped by functional category. (DOC) [file pone.0050417.s001.doc]

**Supporting Information:**

**Table S1:**

| **Biological Function** | **P-Value** | **Gene Name** |
| --- | --- | --- |
| Cellular Growth and Proliferation | 3.92E^-^19-3.15E^-^05 | CRYAB, COL14A1, GLI2, XDH, COL8A1, CCL20, BEX2, IL6, PTPRC, FHL2, SOD2, GAL, LUM, MGP, CCL25, ADORA2B, ITGB4, PRKD1, GUCY1B3, TNFRSF21, IFIT3, LCP1, IL4R, TNFRSF9, PRKCQ, ADD2, HMGA1, IGFBP5, MAFF, TFPI2, CACNA1A, ATP8A2, MET, CCL4, RUNX1T1, THBS2, FADS2, ITGA1, DOK1, TNFAIP6, THRB, ECM1, CTNND2, ICAM1, PHLDA2, OSGIN1, IL1RL1, LRP1B, SEMA6A, MFGE8, CCL5, ITGB8, SLPI, PRKG1, LAMP3, MCF2, DUSP5, GADD45A, C1QTNF3, DPT, PLIN2, CRTC1, DAB2, SERPINA1, LY75, HTR2A, TRAF1, S100A1, CCR1, ITGA2, PTGS1, SERPINF1, RGS16, CCL11, VIP, PLA2G4A, NR4A2, SRGN, ABCB4, NTRK3, LMO3, SERPINH1, PTH1R, LYN, BMP7, DCX, LRRC32, WFDC1, GRIN2A, LOXL4, LIF, MMP3, CDCP1, MBP, S100A4, MEIS1, RARG, GATA2, PVR, GPNMB, GPX3, CXCL3, TNFSF9, ENG, TEK, ITK, TNFRSF11B, TBX5, MTUS1, IL8, ELN, NOX4, MYCT1, ADAMTS1, CNKSR1, THBS1, KCNK2, ORC6L, IL27, CREG1, PTGDS, DCHS1, HDAC5, IGF2, APCDD1, APLN, AKR1B10, PNP, PRKCH, MDK, PPAT, AGTR1, LGR4, DCBLD2, COL8A2, NTF3, YPEL3, MMP7, IL12A, FRZB, MSR1, RECK, NRG1, CCL3, IFITM3, CHRM3, TGFB1I1, DHCR7, SDC2, MCAM, HGF, HS6ST2, ERRFI1, ENPP2, TIMP3, VCAM1, ST8SIA1, EDNRB, SPRY1, UPP1, PLAUR, NFATC4, CALCA, CADM1, TLR4, SHB, TENC1, GHR, SSTR2, LAMA4, HLA-E, NFE2, CALCR, SPARC, WISP1, SCN3B, CXCL2, BDKRB1, C5ORF13, A2M, ISG20, GATA4, IL11, NR6A1, CTGF, AIM2, F2R, |
| Tissue Development | 4.71E^-^18-3.03E^-^05 | PSG2, CRYAB, COL14A1, GLI2, XDH, CCL20, CP, IL6, NPTX1, PTPRC, NCAM2, FHL2, FRAS1, LUM, JPH1, CCL25, VIT, ADORA2B, ITGB4, PRKD1, GUCY1B3, PODXL, TNFRSF9, LCP1, PRKCQ, SEMA5A, SPOCK2, IGFBP5, FMOD, SMOC2, MET, CCL4, THBS2, ITGA1, PLTP, THRB, TNFAIP6, ECM1, CHST6, CTNND2, SERPING1, ICAM1, IGSF1, RSAD2, MFGE8, ITGB8, CCL5, SLPI, EGFLAM, PRKG1, COL16A1, ALX4, DAB2, ABI3BP, CCR1, PTGS1, ITGA2, KAL1, SERPINF1, SALL1, VIP, CCL11, CYTH3, PLA2G4A, NR4A2, MAGI1, EDIL3, NTRK3, PTH1R, LYN, BMP7, SGCD, CSRP3, LIF, MMP3, CDCP1, EDA, CX3CR1, HOXC4, RARG, PVR, GPNMB, CXCL3, GRASP, ENG, TEK, ITK, TNFRSF11B, TBX5, IL8, ELN, NOX4, ADAMTS1, THBS1, IL27, HDAC5, IGF2, RND3, SCARF2, RIPPLY2, CCDC80, LGR4, MMP7, NTF3, IL12A, MSR1, RECK, NRG1, CCL3, TGFB1I1, VNN1, ICAM2, SDC2, OLFML2A, MCAM, HGF, ENPP2, ARC, TIMP3, VCAM1, ST8SIA1, FEZ1, SPRY1, SMOC1, PLAUR, CALCA, CADM1, TLR4, GHR, LAMA4, MNX1, CALCR, PITX1, SPARC, COL11A1, CXCL2, BDKRB1, GATA4, IL11, CTGF, F2R, HSPB2, PCDHGC3, TLX2, CXCL10, ST8SIA4, GAA, COL10A1, PRDM1, NFKBIB, ITGB5, ICOSLG,, PERP, ERBB3, NFASC, TNFRSF11A, IPP, HMGA2, NR2E1 (includes EG:7101), CLDN1, CXCL12, OLFML2B, TGFA, EPHB3, CYP2J2, DSG2 |
| Cell-To-Cell Signaling and Interaction | 5.27E^-^18-3.07E^-^05 | DPYSL2, CSTA, COL14A1, GLI2, ELAVL3, XDH, IL6, BEX2, PTPRC, NCAM2, LHX6, SOD2, FHL2, GAL, IFITM2, LUM, CCL25, MGP, ADORA2B, ITGB4, PRKD1, LCP1, TNFRSF9, IL4R,, RNF112, TRAF1, S100A1, CCR1, DNER, ITGA2, PTGS1, RGS16, SERPINF1, CDO1, EPHA3, CCL11, VIP, PLA2G4A, EDIL3, NR4A2, LMO3, NTRK3, ONECUT2, PTH1R, LYN, BMPER, BMP7, DCX, CSRP3, GRIN2A, LIF, MMP3, CDCP1, POU2AF1, S100A4, NFE2, CD274, HEY2, NFKBIB, AHR, ICOSLG, ITGB5, DMBT1, PDGFRB, SFRP4, CMKLR1, FBLN5, DCN, LAG3, HBEGF, L1CAM, MMP2, IER3, HLA-DQB1, TIMP4, CCND2, ADRB1, TNFSF12, LZTS1, HOXA11, GAS1, MAL, RARB, CEBPA, BTG4, HRK, FGF7, IL11RA, CTSK, RGS2, ENPP1, MREG, BHLHE22, SLIT2, ITGA3, AVIL, COL5A3, SOX6, BMF, PLXNB1, MSTN, CHRDL1, CSF2, LRP1, HOXB5, GAS7, TFF3, PLP1, INSIG1, IL32, CXCL1, PBX1, ACVRL1, SDC4, NID1, NAP1L2, VASH1, TOP1, TNN, ITGA11, ACSL5, POSTN, NEO1, LHX8, PRLR, ELOVL7, DSP, RIPK4, GJA1, COL4A1, SPP1, FST, NOTCH3, BCAR3, GSN,, KIFAP3, HMGA2, COL1A1, NR2E1 (includes EG:7101), EBF1, CXCL12, CRABP2, LINGO1, TGFA, EPHB3, PRDM16, VGF |
| Cellular Development | 5.93E^-^15-3.72E^-^05 | DPYSL2, CSTA, COL14A1, GLI2, ELAVL3, XDH, IL6, BEX2, PTPRC, NCAM2, LHX6, SOD2, FHL2, GAL, IFITM2, LUM, CCL25, MGP, ADORA2B, ITGB4, PRKD1, LCP1, TNFRSF9, IL4R, PRKCQ, ADD2, ACAN, HMGA1, BMP7, DCX, CSRP3, GRIN2A, LIF, MMP3, CDCP1, MBP, POU2AF1, S100A4, MEIS1, RARG, GATA2, PVR, CXCL3, CCL8, ENG, TEK, TNFRSF11B, ITK, TBX5, IL8, NOX4, MYCT1, CNKSR1, THBS1, CAND2, IL27, CREG1, PTGDS, HDAC5, IGF2, RND3, APCDD1, APLN, PNP, PRKCH, MDK, CCDC80, AGTR1, DOK5, PTGFR, MMP7, GATA5, NTF3, IL12A, FRZB, MSR1, NRG1, CCL3, DHCR7, TGFB1I1, ICAM2, VNN1, SDC2, MCAM, HGF, TIMP3, VCAM1, AGFG1 (includes EG:3267), DCN, LAG3, HBEGF, L1CAM, MMP2, TIMP4, CCND2, HSPG2, MSC, EMILIN2, FN1, PTN, PARD6B, COL4A2, SDC3, MEOX2, CDH11, VCAN, SELENBP1, HOXB5, GAS7, TFF3, PLP1, INSIG1, IL32, CXCL1, PBX1, ACVRL1, SDC4, NID1, NAP1L2, VASH1, TOP1, TNN, ITGA11, ACSL5, POSTN, NEO1, LHX8, PRLR, ELOVL7, DSP, RIPK4, GJA1, COL4A1, NOTCH3, BCAR3, GSN, IL24, SMARCD3, MLPH, TRAPPC9, NANOG, COL6A3, IL1RN, PAX3, GNAO1, S100B, PECAM1, FGF5, PDPN, SH2B3, ADAMTS7, SFRP2, GDNF, PTGER3, PRDM16, VGF |
| Organismal Development | 3.52E^-^13-2.88E^-^05 | NR6A1, CRYAB, GLI2, CTGF, F2R, IL6, NPTX1, PCOLCE, CXCL10, ST8SIA4, CTSS, GAA, LUM, PRDM1, ADORA2B, HEY2, CALB1, ITGB5, AHR, LHX9, DMBT1, PDGFRB, PDE2A, GUCY1A3, FBLN5, SEMA5A, ZC3HAV1, HBEGF, IGFBP5, MMP2, L1CAM, FMOD, SMOC2, MAFF, MET, CCL4, CCND2, ITPR3, THBS2, FADS2, ITGA1, THRB, ECM1, EPHA2, COL3A1, HIST1H1C, APBA2, HTATIP2, FN1, ICAM1, CUBN, MFGE8, SERPINH1, PTH1R, PLXNB1, NR5A2, BMP7, COL15A1, CSF2, LRP4, FOXP2, LIF, MMP3, PBX1, CXCL1, S100A4, ACVRL1, MEIS1, CX3CR1, RARG, GATA2, ABCA1, CXCL3, VASH1, ACSL5, CXCL14, POSTN, PRLR, ENG, ARHGAP24, TEK, TNFRSF11B, TBX5, NPY, ELN, GCH1, IL8, TTR, GJA1, FST, ADAMTS1, THBS1, AQP1, CREG1, GSN, MC4R, IL24, HDAC5, OGN, CRHBP, IGF2, NANOG, IL1RN, PAX3, LFNG, MDK, AGTR1, FGF5, MDFI (includes EG:4188), SH2B3, SFRP2, IL12A, PTGER3, RECK, NRG1, CLU, TNFAIP3, ING4, HIF1A, CCL3, CHRM3, HOXB13, ROR2, HMOX1, DHCR7, NFKBIA, ANGPTL4, GLI3, CSPG4, SP8, ERBB4, HGF, KIT, MMP11, SEMA3E, TIMP3, VCAM1, S1PR2, EFEMP2, C2CD3, PLAUR, TNFRSF11A, HMGA2, CADM1, COL1A1, TLR4, GHR, SLC14A1, LAMA4, NFE2, CXCL12, CRABP2, PITX1, TGFA, SPARC, EPHB3, GATA4, IL11 |
| Organ Development | 4.81E^-^12-1.26E^-^05 | CRYAB, GLI2, CTGF, COL8A1, IL6, ADAMTS2, SOD2, LHX6, ST8SIA4, PEG10, GAL, RLN1, GAA, FRAS1, MGP, PRDM1, HEY2, AHR, LHX9, PDGFRB, IL4R, PODXL, DCN, HBEGF, TLL1, NR1H3, IGFBP5, MMP2, MET, CCND2, HSPG2 (includes EG:3339), THBS2, ITGA1, THRB, MAB21L1, ALK, COL3A1, ICAM1, FN1, PHLDA2, MEOX2, PRKG1, VCAN, COL5A1, EFNB2, ADRB1, DPT, HOXA11, RARB, GAS1, CEBPA, PDGFRA, FGF7, IL11RA, S100A1, ITGA2, SERPINF1, SALL1, SLIT2, ITGA3, COL5A3, ONECUT2, NTRK3, BMF, PTH1R, BMP7, CSF2, DCX, FOXP2, LRP4, DNMT3A, EDA, PBX1, AFF2, HOXC4, GATA2, RARG, ABCA1, NPNT, CXCL3, TNFSF9, OSR1, LHX8, PRLR, ENG, DSP, TBX5, ELN, GJA1, NOTCH3, FST, OVOL2, ADAMTS1, THBS1, IL27, GSN, RBMY1B, SMARCD3, HDAC5, IGF2, APLN, PAX3, FBN1, LFNG, AGTR1, LGR4, PDPN, SH2B3, COL8A2, NTF3, GDNF, TLE2, MAPT, NRG1, TNFAIP3, TTPA, HIF1A, HOXB13, DHCR7, AEBP1, GLI3, HGF, GFRA1, SOX3, ERBB4, SEMA3E, COL5A2, VCAM1, EDNRB, SPRY1, ZIC1, C2CD3, NFATC1, ERBB3, NFATC4, TNFRSF11A, CADM1, TLR4, COL1A1, TENC1, NR2E1 (includes EG:7101), LAMA4, CLDN1, CXCL12, MNX1, PITX1, TGFA, BIK, SPARC, COL11A1, GATA4, IL11 |
| Nervous System Development and Function | 1.79E^-^09-3.47E^-^05 | DPYSL2, XDH, IL6, NPTX1, PTPRC, LHX6, GAL, GFAP, ITGB4, PDGFRB, SEMA5A, ACAN, NR1H3, HBEGF, SPOCK2, L1CAM, MMP2, CACNA1A, MET, PEX2, ITGA1, THRB, REG3G, EPHA2, TMEM158, ALK, CTNND2, APBA2, CLDN11, FN1, PTN, CCL5, SDC3, PRKG1, VCAN, EFNB2, FGF7, IL11RA, CCR1, DISC1, DNER, SERPINF1, SLIT2, VIP, ITGA3, NR4A2, NTRK3, BMP7, CSF2, SYNGR1, DCX, GAS7, PLP1, LIF, SERPINI1, UNC5B, CXCL1, S100A4, SCN4B, CXCL3, SEMA3D, GJA1, THBS1, STK38L (includes EG:23012), DPYSL5, GSN, HDAC5, IGF2, S100B, GNAO1, MDK, FGF5, DOK5, NTF3, GDNF, NRG1, MAPT, NFKBIA, GLI3, HGF, GFRA1, ERBB4, PTPRZ1, KIT, ERRFI1, ADRB2, VCAM1, EDNRB, FEZ1, PLAUR, NFATC4, EBF1, LAMA4, CXCL12, MNX1, LINGO1, TGFA, CXCL2, A2M, IL11 |
| Embryonic Development | 1.81E^-^09-3.74E^-^05 | LIF, XDH, CCL20, IL6, POSTN, CCL8, PRLR, ENG, SAA1, ITGB5, TEK, TNFRSF11B, PDGFRB, SFRP4, NOX4, C3, OVOL2, SPP1, ADAMTS1, THBS1, ACAN, IL27, HBEGF, IGFBP5, MMP2, GSN, MC4R, SPRR2A, IGF2, IL1RN, PAX3, LFNG, ITGA1, PDGFD, EPHA2, ECM1, CA2, SFRP2, FN1, ICAM1, MSR1, GDNF, NRG1, MEOX2, HIF1A, CCL5, TTPA, CCL3, VCAN, HMOX1, EFNB2, TNFSF12, ERBB4, HGF, SPRR2C, ENPP2, ADRB2, IL11RA, CCR1, TIMP3, CTSK, VCAM1, ENPP1, EDNRB, ITGA2, PLAUR, SPRR2D, SLIT2, TNFRSF11A, VIP, ITGA3, CALCA, EDIL3, CXCL12, PTH1R, CALCR, SPARC, EPHB3, BMP7, GATA4, SPRR2E, IL11 |
| Organismal Survival | 9.89E^-^09-9.22E^-^07 | NR6A1, F2R, IL6, TLX2, CXCL10, PTPRC, ST8SIA4, SOD2, RLN1, KIF1A, ADORA2B, CD274, HEY2, ITGB4, NFKBIB, AHR, DMBT1, PDGFRB, PTGIS, IL4R, TNFRSF9, PODXL, DCN, TLL1, HBEGF, MMP2, IGFBP5, IRF5, MAFF, CACNA1A, UBD, MET, NOD2, HSPG2, CCND2, MME, SERPING1, ICAM1, IL1RL1, ITGB8, COL4A2, SLPI, DMD, PRKG1, COL5A1, EFNB2, ADRB1, GADD45A, PDGFRA, FGF7, IL11RA, S100A1, CCR1, PTGS1, DGAT2, CCL11, VIP, F3, NR4A2, ABCB4, NTRK3, PTH1R, LYN, NR5A2, BMP7, CSF2, FOXP2, LIF, DNMT3A, EPHX2, PBX1, CX3CR1, SDC4, ABCA1, NAP1L2, CXCL3, TOP1, TNFSF9, KRT18, POSTN, ENG, DSP, TEK, ITK, TNFRSF11B, FST, C3, COL4A1, SGCG, AQP1, IL27, IGF2, IL1RN, PAX3, PNP, FBN1, CCL3L3, MDK, IL12A, GDNF, MSR1, RECK, NRG1, TNFAIP3, HIF1A, TTPA, HMOX1, AEBP1, GABRB3, NFKBIA, HGF, ERBB4, KIT, ENPP2, HSPB6, CXCL11, ADRB2, TIMP3, VCAM1, ST8SIA1, S1PR2, EFEMP2, BGN, ZIC1, PERP, NFATC1, ERBB3, KIFAP3, HMGA2, TLR4, SLC14A1, CLDN1, CXCL12, NFE2, CRABP2, TGFA, EPHB3, CXCL2, BDKRB1, GATA4, IL11 |
| Ophthalmic Disease | 5.54E^-^07-5.54E^-^07 | CRYAB, GRIN2A, C1QTNF5, CP, IL6, CX3CR1, GPNMB, MAOB, FRAS1, POSTN, GFAP, SAA1, CHRM2, PDGFRB, IL8, C3, FBLN5, THBS1, DCN, MMP2, HMCN1 (includes EG:83872), IL1RN, LOXL1, FBN1, CCL3L3, PECAM1, AKR1B1, CHST6, COL8A2, PTGFR, CA2, PTGER3, CLU, TTPA, CCL5, CHRM3, VCAN, ADRB1, HGF, PDGFRA, KIT, CFH, CHRNA5, ADRB2, HTR2A, SCN1A, FREM2, VCAM1, EDNRB, EFEMP2, PTGS1, RP1, SERPINF1, PLAUR, TLR4, NR2E1 (includes EG:7101), TGFA, FBLN2, COL11A1, EFEMP1 |
| DNA Replication, Recombination, and Repair | 5.55E^-^07-5.55E^-^07 | COL14A1, GLI2, LIF, F2R, IL6, SDC4, CXCL10, ADORA2B, PRKD1, GUCY1B3, IL8, GJA1, SPP1, FBLN5, GUCY1A3, HBEGF, IGFBP5, MMP2, PTGDS, SMOC2, TFPI2, IGF2, CCND2, IL1RN, GNAO1, THBS2, PDGFD, PPAT, AGTR1, ALK, PTGFR, NTF3, FN1, GDNF, PTN, NRG1, CLU, CHRM3, NFKBIA, HGF, ERBB4, PDGFRA, CEBPA, SERPINA1, ERRFI1, SALL2, FGF7, S1PR2, ERBB3, CALCA, NTRK3, CXCL12, TGFA, SPARC, BMP7, EFEMP1, CSF2, A2M |
